# Direct imaging of the recruitment and phosphorylation of S6K1 in the mTORC1 pathway in living cells

**DOI:** 10.1038/s41598-019-39410-z

**Published:** 2019-03-04

**Authors:** Abdullah R. Ahmed, Raymond J. Owens, Christopher D. Stubbs, Anthony W. Parker, Richard Hitchman, Rahul B. Yadav, Maud Dumoux, Chris Hawes, Stanley W. Botchway

**Affiliations:** 1 Central Laser Facility, Research Complex at Harwell, STFC Rutherford Appleton Laboratory, Harwell Campus, Didcot, OX11 0FA UK; 2 Evotec (UK) Ltd, 114 Innovation Drive, Milton Park, Abingdon, Oxfordshire OX14 4RZ UK; 3 Protein Production UK, Research Complex at Harwell, Rutherford Appleton Laboratory, Harwell Campus, Didcot, OX11 0FA UK; 4 The Wellcome Centre for Human Genetics, Roosevelt Drive, Oxford, OX3 7BN UK; 5 Diamond Light Source, Harwell Campus, Didcot, OX11 0DE UK; 6 Oxford Brookes University, Headington Campus, Oxford, OX3 0BP UK

## Abstract

Knowledge of protein signalling pathways in the working cell is seen as a primary route to identifying and developing targeted medicines. In recent years there has been a growing awareness of the importance of the mTOR pathway, making it an attractive target for therapeutic intervention in several diseases. Within this pathway we have focused on S6 kinase 1 (S6K1), the downstream phosphorylation substrate of mTORC1, and specifically identify its juxtaposition with mTORC1. When S6K1 is co-expressed with raptor we show that S6K1 is translocated from the nucleus to the cytoplasm. By developing a novel biosensor we demonstrate in real-time, that phosphorylation and de-phosphorylation of S6K1 occurs mainly in the cytoplasm of living cells. Furthermore, we show that the scaffold protein raptor, that typically recruits mTOR substrates, is not always involved in S6K1 phosphorylation. Overall, we demonstrate how FRET-FLIM imaging technology can be used to show localisation of S6K1 phosphorylation in living cells and hence a key site of action of inhibitors targeting mTOR phosphorylation.

## Introduction

The mammalian Target of Rapamycin (mTOR) pathway has a vital role in the co-ordination of energy, nutrients and growth factor availability to regulate key biological processes including cellular growth, metabolism and protein synthesis through the phosphorylation of downstream ribosomal protein, S6 Kinase 1 (S6K1)^1^. S6K1 also functions in cell structure and organisation^2^, has been shown to regulate aging and adiposity^3^, memory^4^, immunity^5^ and muscle hypertrophy^6^.

The growing importance of mTOR is emphasized by the considerable body of research that has been produced within the last decade. Of particular note is the belief that the mTOR signalling pathway provides a means to treat numerous diseased states and this has driven extensive studies investigating how dysfunctional mTOR signalling can lead to cancer, type II diabetes, cardiovascular and neurological diseases^7,8^. Human mTOR works in concert and is part of a multi-protein complex with Rheb, raptor, mLST8, PRAS40 and DEPTOR proteins to create the mTOR Complex 1 (mTORC1). Assembly of mTORC1 is currently thought to phosphorylate the substrate S6K1 for normal cellular function. Furthermore, a second mTOR complex may also contain rictor, Protor, mLST8, Sin1 and DEPTOR proteins to form mTOR Complex 2 (mTORC2)^9^. Increasing our understanding of the mTOR complex proteins and their physical interactions, where within the cell these assemblies are localised and where subsequent phosphorylation of downstream targets occur, is seen as key to developing new drug targets. To date we find no evidence implicating mTORC2 functioning via phosphorylation of S6K1^10^. This work therefore specifically focusses on the recruitment and localisation of the mTORC1 complex and phosphorylation of S6K1 in live cells.

A vital step towards the development and optimisation of drugs is a need to understand the localisation of both the cell target (subcellular), visualisation of the drug and how they interact within a nominated cellular pathway in real time. A possible strategy to inhibit the mTOR activity is to restrain S6K1 phosphorylation and to do this, requires understanding of where S6K1 is found within the cell with respect to the mTOR complex as well as the key drivers in its phosphorylation. Within the working cell, S6K1 has been reported to be located in a variety of cellular compartments. Observations made from cell fractionation studies have indicated the presence of S6K1 both in the cytoplasm and the nucleus^11,12^. More recently, work with fixed cells suggests only a cytoplasmic localisation^13^ and the only recorded live imaging has been performed in plant cells, using GFP-S6K1^14^ which showed a nucleocytoplasmic localisation of S6K1. Nuclear localisation has further been shown by the use of immunofluorescence labelling studies^15^. Although S6K1 exists in multiple isoforms (produced from the RPS6KB1 gene due to an alternative start and alternative splicing codons), only two are targets for mTOR phosphorylation, with threonine residue389 on p70 S6K1 and threonine residue412 on p85 S6K1 isoforms. Thus, whilst S6K1 appears to be widely distributed within cells, determining the specific location of phosphorylated S6K1 in cells remains a key issue in relation to the mTOR pathway.

Identifying where S6K1 phosphorylation occurs has been approached in a variety of ways, mainly indirect, and cell fractionation work by Rosner and Hengstschläger indicates phosphorylation of p70 S6K1 isoform causes the translocation of S6K1 from the cytoplasm into the nucleus^11^, although the mechanism of this process is unknown. Other S6K1 phosphorylation studies, using fixed cell immunofluorescence labelling for phospho-S6K1 upon amino acid activation^16^, support the findings from Rosner and Hengstschläger, although the drivers for the migration of the phosphorylation components are unknown. A much needed method to monitor phosphorylation would be the ability to perform observations in living cells in real-time and overcoming the well-known problems with cell fixation. Recently, S6K1 has been reported to undergo a conformational change upon phosphorylation as evident by linking mutations and truncations of S6K1 to its activation^17^. We believe this structural change in S6K1 may provide a means to image directly the phosphorylation process using Fluorescence Resonance Energy Transfer (FRET)/GFP technology.

To achieve this goal we have produced a novel S6K1 sensor of mTOR (SensOR) which upon phosphorylation results in a change in fluorescence lifetime that can be monitored using the FRET-FLIM (Fluorescence Lifetime Imaging Microscopy) method. Our current work improves upon previous studies on live cells that used a similar approach to observe mTOR phosphorylation using the FRET sensor (Eevee-S6K) to indirectly report on mTOR phosphorylation^18^ by monitoring rictor phosphorylation by S6K1^19^.

Two major components of the pathway are the Rheb and raptor proteins. The Rheb GTPase protein is suggested to activate mTORC1 in the GTP bound state and association with mTOR^10,18,20–24^ on the lysosomal membrane^25^. The raptor protein is reported to act as a ‘scaffold’ due to its ability to bind to mTORC1 substrates, S6K1 and 4EBP1^26^ as well as the inhibitory protein PRAS40^27^. To date most of the work investigating S6K1 and raptor interactions in both mammalian and plant cells has been *in vitro*, typically consisting of pull-down assays^14,26^.

Our earlier work showed raptor to be located primarily within the cytoplasm^21,28^ but the mechanism behind the recruitment of the substrates directly onto mTOR remains unknown and drives a second important question, namely to establish the subcellular localisation and factors that promote assembly of the various mTOR components within a common cytoplasmic region where phosphorylation of S6K1 occurs. Tracking of S6K1 can be performed using GFP technology as well as modifications to the protein, e.g. with a mutated TOR Signalling (TOS) motif, on the N-terminus^29,30^. The TOS motif mediates the interaction with raptor as mutations in the S6K1 TOS sequence abolishes binding as shown by pull-down^31^. Other mTOR substrates such as 4EBP1 and PRAS40 also have unique TOS motifs, allowing them to bind to raptor^32–36^. An alternative strategy to determine the significance of the observed assembly of the complex (mTORC1) and substrate is to utilise the competitive nature of S6K1, 4EBP1 and PRAS40 for the same binding site on raptor^37,38^.

We report the application of advanced fluorescence imaging methods (FRET-FLIM) using a series of fluorescently labelled mTORC1 proteins to gain information on the recruitment of the downstream mTOR target, S6K1. We have specifically determined its subcellular localisation and the relationship between S6K1 and mTORC1 in living cells as well as the ultimate sub-cellular phosphorylation sites. The latter has been imaged directly using a novel S6K1 biosensor which demonstrates how FRET-FLIM technology can be used to report directly on active and real-time cell signalling, providing the opportunity for high-throughput mTOR assays for drug discovery.

## Results

### S6K1 nucleocytoplasmic localisation in live cells

**Figure 1:**
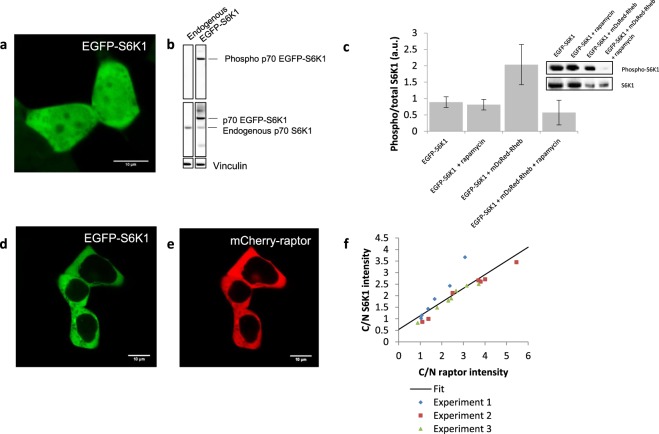
S6K1 live cell localisation and recruitment onto mTORC1 in HEK293 cells. (**a**) Confocal image of EGFP-S6K1 only. (**b**) Western blot validation of the functionality of the EGFP-S6K1 construct. (**c**) Graph and blot showing S6K1 phosphorylation with and without Rheb co-expression +/− rapamycin (200 nM) treatment. Data taken from Western blot of HEK293 cells and bands quantified using densitometry analysis (ImageJ software 1.48 V), ratio = Phospho-EGFP-S6K1/EGFP-S6K1 where EGFP-S6K1 band correlates to both phosphorylated and unphosphorylated. (**d**,**e**) Confocal images of EGFP-S6K1 with mCherry-raptor co-expression. (**f**) Graph of fraction of mean cytoplasmic/nuclear S6K1 intensities against fraction of mean cytoplasmic/nuclear raptor intensities (C/N) with linear fit. Data representative of three independent experiments with errors representative of standard deviation. Full-length blots are presented in Supplementary Fig. S10 for (**b**) and in Supplementary Fig. S13 for (**c**).

In order to determine the subcellular localisation of S6K1, we genetically fused the N-terminus of S6K1 to the C-terminus of EGFP and expressed the construct in live HEK293 cells (Fig. 1a). Western blot analysis showed that the EGFP-S6K1 construct was functional as evident by the generation of phospho-EGFP-S6K1 by endogenous mTOR (Fig. 1b). EGFP-S6K1 was also able to phosphorylate its own substrate, RPS6. Using confocal microscopy, we observed EGFP-S6K1 localisation in both the cytoplasm and in the nucleus in near equal amounts with 54% EGFP intensity in the cytoplasm and 46% intensity in the nucleus (10% standard deviation) (Fig. 1a). We note that basal endogenous levels of phospho-S6K1 proved difficult to detect, most likely due to its low levels in HEK293^34,39^ (Fig. 1b)), reduced sensitivity of the antibodies used as well as loss of cytoplasmic proteins due to current fixation methods such as paraformaldehyde and methanol. C-terminal fluorescently tagged S6K1 constructs were also made and expressed in HEK293 cells. S6K1-mTurqouise2 and S6K1-mCherry both showed similar subcellular localisation to EGFP-S6K1 (Supplementary Fig. S1a).

### Key role of Rheb in S6K1 phosphorylation and dephosphorylation by rapamycin

Using transient overexpression of proteins as a “test-tube” approach to study mTORC1 regulation, fluorescently tagged Rheb was co-expressed with EGFP-S6K1 and examined by Western blot. As shown in Fig. 1c, S6K1 phosphorylation was dramatically increased by Rheb co-expression by more than a factor of two. More importantly, phosphorylation was reduced (~50%) by treatment with 200 nM rapamycin, as shown by densitometry analysis of Western blots, but only in the presence of Rheb. This indicates that Rheb is directly involved in the inhibition of mTOR phosphorylation activity by rapamycin. However, we have not investigated the possible interplay between Rheb and FKBP12 (the 12-kDa FK506-binding protein) which is the main rapamycin binding site on the mTORC1. The regulation of S6K1and its phosphorylation by other mTORC1 subunits, such as Rheb, suggests that mTOR may not be the key ‘rate limiting’ protein in mTORC1 signalling. The augmented inhibition of S6K1 phosphorylation by over expression of Rheb suggests that there is a need to target Rheb as well as mTOR to regulate the pathway in clinical applications.

### S6K1 dynamics with mTOR, raptor and Rheb proteins as well as their physical interaction in living cells

We investigated protein-protein interactions within the mTORC1 pathway using FRET-FLIM technology by co-expressing Rheb, raptor and mTOR together with EGFP-S6K1. Firstly, EGFP-S6K1 was expressed and imaged with mCherry-raptor in live HEK293 cells to investigate the relationship between S6K1 and raptor as the scaffold protein for the complex. Co-expression of the two mTORC1 proteins led to S6K1 becoming predominately localised in the cytoplasm, similar to raptor (Fig. 1d,e) and was consistent with other fluorescently tagged raptor and S6K1 constructs (Supplementary Fig. S1a,b). This change in localisation, referred to as translocation, was generally similar to results where YFP or mCherry was tagged to raptor. The level of S6K1 translocation appeared to be proportional to the expression level of raptor such that high raptor expression in the cytoplasm caused higher amounts of S6K1 to be translocated out of the nucleus. Thus, the amount of S6K1 present within the cytoplasm was directly proportional to the expression levels of raptor (Fig. 1f). This translocation effect may represent the first steps in S6K1 recruitment onto mTORC1 in live cells by raptor.

**Figure 2:**
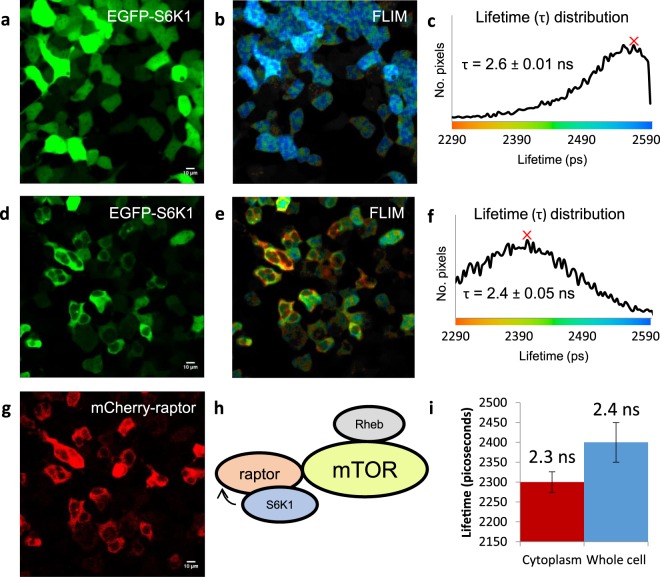
Live cell interaction between S6K1 and raptor using FRET-FLIM in HEK293 cells. (**a**) Confocal image of EGFP-S6K1. (**b**,**c**) FLIM of EGFP-S6K1 with corresponding lifetime distribution histogram showing lifetime (τ). (**d–g**) Confocal images of EGFP-S6K1 with mCherry-raptor co-expression with FLIM and lifetime distribution histogram. (**h**) Schematic showing summary of interaction. (**i**) Graph showing cytoplasmic against whole cell lifetimes between EGFP-S6K1 and mCherry-raptor, selected by selecting subcellular regions and obtaining the mode lifetime in SPCImage V6.0. Data representative of three independent experiments with errors representative of standard deviation.

Having confirmed co-localisation of S6K1 with raptor, we went on to investigate whether these two proteins have a direct physical interaction in live cells using FRET-FLIM imaging of EGFP-S6K1 (donor) and mCherry-raptor (acceptor). Prior to the FLIM data acquisition, cells expressing equal levels of both the donor and acceptor or higher level of the acceptor than the donor were selected using the carefully calibrated confocal laser scanning microscopy^40^. The excited state fluorescence lifetime of EGFP-S6K1 alone was found to be 2.6 ± 0.01 ns and this was reduced to 2.4 ± 0.05 ns overall in the presence of mCherry-raptor, with higher quenching to 2.3 ± 0.03 ns specifically in the cytoplasm (Fig. 2c,f,i). The lower lifetime observed in the cytoplasm compared to that in the nucleus may be due to the higher levels of mCherry-raptor present in the cytoplasm following translocation. We sought to fit our observed fluorescence lifetime distribution to a known function. Several functions were attempted but the simplest approach to lifetime distributions assumes that the function f(r) to be uniform, i.e. Gaussian or Lorentzian. The kinetic profile fitted well to a Gaussian distribution – typical of a natural probability function and this enabled a statistical measure to be made identifying (Supplementary Fig. S2a) a significant difference between EGFP-S6K1 alone and in the presence of mCherry-raptor. This key result not only supports previous pull-down studies^31^ that demonstrated S6K1-raptor association but importantly identifies a direct physical interaction between S6K1 and raptor in live HEK293 cells (Fig. 2). It is worth noting that in certain conditions, a free-floating mCherry -not tagged to any protein, may cause a small non-specific binding with other proteins due to random localisation. However, this is not the case when the mCherrry is tagged to a specific protein and thus have a specific localisation.

The distribution of S6K1 remained approximately equal between cytoplasm and nucleus when co-expressed with fluorescently tagged Rheb or mTOR constructs, though S6K1 co-localised in the cytoplasm was more pronounced with mTOR than Rheb (Supplementary Figs S3 and S4). FRET-FLIM studies of EGFP-Rheb or EGFP-mTOR (donors) with S6K1-mCherry (acceptor) did not show a strong direct interaction. A slight quenching from 2.6 ± 0.01 ns (EGFP-mTOR lifetime) to 2.5 ± 0.03 s in the presence of S6K1-mCherry and no change in lifetime of EGFP-Rheb with S6K1-mCherry was observed (Supplementary Figs S3 and S4). The small quenching with mTOR may suggest S6K1 is weakly interacting with mTOR or that the interaction may be one of a dynamic nature. As further validation for our findings, pull-down studies of EGFP-S6K1 (containing an N-terminus His tag) with mCherry-raptor and/or with FLAG-mTOR or with mDsRed-Rheb were performed using nickel binding resins. As shown in Supplementary Fig. S5, EGFP-S6K1 was pulled-down with raptor, or with mTOR (but it is also possible that the presence of endogenous raptor may be bridging the two proteins together) and no pull-down with Rheb was observed. Collectively, the pull-down data are consistent with our findings from the FRET-FLIM results.

### Inhibition of S6K1 translocation by S6K1 TOS motif mutation or by PRAS40 and 4EBP1

Having established S6K1 is bound to the complex, we proceeded to characterise the mechanism of EGFP-S6K1 translocation into the cytoplasm and if this involved a direct interaction with raptor. The TOR signalling (TOS) motif in the N terminus of S6K1 has been implicated in the interaction with raptor in pull-down experiments and is disrupted by mutating F28A^31^. This mutation was introduced into the EGFP-S6K1 and on co-expression with raptor was shown not to be re-distributed in living cells (Supplementary Fig. S6), indicating that the TOS motif is required for the recognition and recruitment of S6K1 by raptor and its subsequent re-distribution in live cells.

We then investigated whether inhibiting phosphorylation of S6K1 would affect its translocation to the cytoplasm by co-expressing S6K1 and raptor with either PRAS40 or 4EBP1 proteins which are known to inhibit S6K1 phosphorylation. YFP-PRAS40 was overexpressed with S6K1-mTurqouise2 and mCherry-raptor^27^. The translocation of S6K1 with raptor was inhibited as shown in Supplementary Fig. S6. S6K1 was unable to translocate in the presence of PRAS40. Interestingly, GFP tagged 4EBP1 (4EBP1-GFPSpark) also translocated with mCherry-raptor co-expression (Supplementary Fig. S7), indicating that raptor’s substrate recognition and recruitment role in the complex extends to all mTOR substrate targets investigated here. A similar inhibition of S6K1 translocation was seen from preliminary data of 4EBP1-GFPSpark co-expression with both S6K1-mTurqouise2 and mCherry-raptor (Supplementary Fig. S7). These results indicate that translocation of S6K1 to the cytoplasm may be a vital pre-requisite for phosphorylation.

### Role of mTOR in the recruitment of S6K1 onto the mTORC1 complex

**Figure 3:**
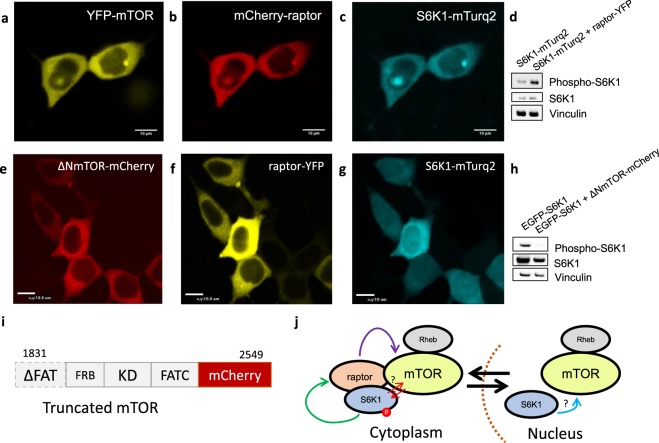
mTOR mediated recruitment of S6K1 onto mTORC1 in the cytoplasm. (**a–c**) Confocal images of wildtype YFP-mTOR, mCherry-raptor and S6K1-mTurquoise2 triple-colour expression in live HEK293 cells. (**d**) Western blot for S6K1 phosphorylation (phospho-S6K1) with raptor and S6K1 expression. (**e–g**) Confocal images of truncated mTOR-mCherry (ΔNmTOR-mCherry), raptor-YFP and S6K1-mTurquoise2 expression. (**h**) Western blot for S6K1 phosphorylation with truncated mTOR-mCherry and S6K1 expression. (**i**) Truncated mTOR-mCherry construct showing domains and numbered amino acid sequence. (**j**) Schematic of possible sequence of recruitment and assembly of mTORC1. Data representative of three independent experiments with errors representative of standard deviation. Full-length blots are presented in Supplementary Fig. S13.

Considering raptor has not been observed within the cell nucleus, we asked whether raptor or another protein was involved in sensing S6K1 in the nucleus and responsible for the translocation of S6K1 to the cytoplasm. As there is significant amount of mTOR in the nucleus^21^, we considered whether an as yet unidentified ‘sensing mechanism’ involving mTOR was involved. Thus, a non-functional N-terminal truncated mTOR-mCherry construct was made and expressed with raptor-YFP and S6K1-mTurqouise2 (Fig. 3). The truncated mTOR-mCherry construct inhibited the S6K1 translocation, observed with both raptor and wild-type expression, indicating that fully functioning and assembled mTORC1 is required in this recruitment process. Furthermore, raptor expression with S6K1 (translocated) induced phosphorylation while truncated mTOR-mCherry inhibited S6K1 phosphorylation, indicating a direct link between mTOR assisted translocation, possibly phosphorylation and the localisation of S6K1 (Fig. 3).

### Loss of S6K1 protein and localisation in fixed cells

We attempted to quantify phosphorylation levels in endogenous and transfected cells using cell fixation (methanol and paraformaldehyde) followed by immunofluorescence labelling for phospho-S6K1 (pS6K1). We identified loss of soluble EGFP-S6K1 and consequently found more nuclear S6K1 localisation as well as movement of protein into the nucleolus (Supplementary Fig. S8a,b). Some cells failed to label properly and this could have been due to the fixation process (Supplementary Fig. S8b,c). Furthermore, labelled cells for phospho-S6K1 with translocated EGFP-S6K1 (co-expression with raptor) showed that the translocated EGFP-S6K1 may be phospho-EGFP-S6K1, indicating it may be already phosphorylated in the cytoplasm (Supplementary Fig. S8e–g). EGFP-S6K1 transfected cells were also fixed with methanol and showed loss of EGFP-S6K1 from the nucleus and a more peri-nuclear/ ER localised appearance (Supplementary Fig. S8h). Similarly, immunofluorescence labelling of phospho-S6K1 showed inconsistent results. We conclude that the current fixation methods are insufficient for studying the mTOR signally pathway and that live cell imaging is essential.

### Live cell cytoplasmic localisation of phosphorylated S6K1; the application of a new FRET molecular sensor

**Figure 4:**
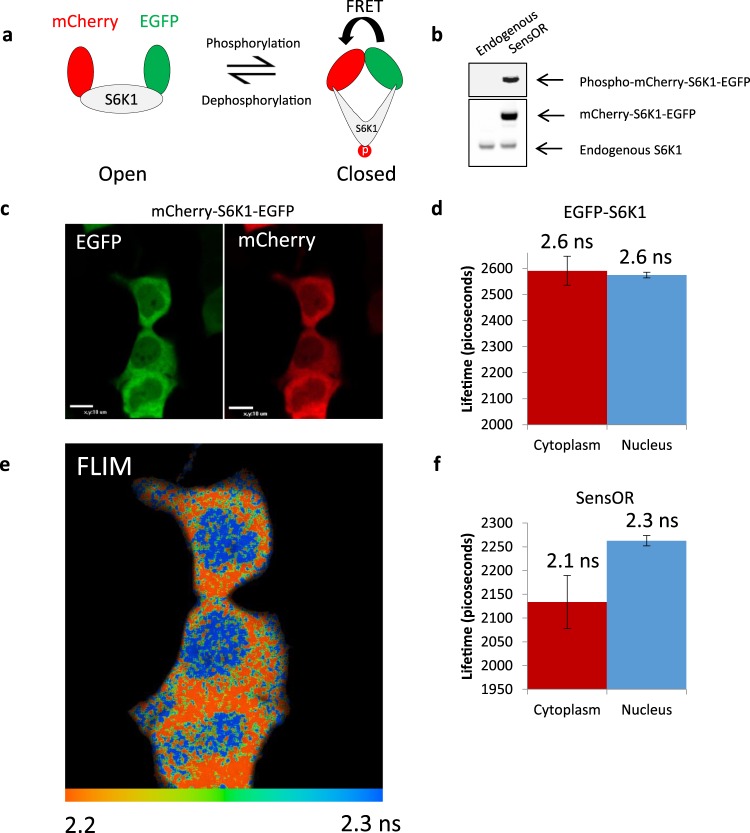
Localisation of phospho-S6K1 in living cells using SensOR. (**a**) Schematic diagram showing conformational changes in the biosensor to induce FRET. (**b**) Western blot for SensOR phosphorylation by endogenous mTOR. (**c**) Confocal images of biosensor alone in HEK293 cells. (**d**) Cytoplasmic versus nuclear lifetime distributions of EGFP-S6K1. (**e**,**f**) FLIM of SensOR with lifetime scale bar below in nanoseconds (ns) and graph showing cytoplasmic against nuclear lifetime distributions of SensOR, selected by selecting sub-cellular regions and obtaining the mode lifetime in SPCImage V6.0 software. Data representative of three independent experiments with errors representative of standard deviation. Full-length blots are presented in Supplementary Fig. S14.

Given the issues with ascertaining levels of phosphorylation in fixed cells and the fact that there are currently no methods to quantify levels of S6K1 phosphorylation in live cells, we introduce a new method to quantify phosphorylation in live cells based on the development of a molecular S6K1 FRET bio-sensor. This novel S6K1 sensor of mTOR (SensOR) enables direct reporting on mTORC1 activity (Fig. 4). Tandem labelled mCherry-S6K1-EGFP was made and expressed in HEK293 cells. FRET-FLIM showed that phosphorylated S6K1 existed in a closed conformation in the cytoplasm (shorter lifetime) and in an open state in the nucleus (longer lifetime) (Fig. 4). Advantageously, we found that highly expressing SensOR cells displayed some translocation of the S6K1 tandem construct which we suspect is due to cells that are in a hyperphosphorylated state. FLIM of these cells, i.e. expression the SensOR (determined from three different experiments) showed distinct lifetimes between the cytoplasm and nucleus (2.1 ± 0.06 ns vs 2.3 ± 0.01 ns (Fig. 4f, error calculated as the standard deviation from n >40 cells from three independent experiments) while no difference was found with the nuclear and cytoplasmic lifetimes of EGFP-S6K1 (Fig. 4d). Here we speculate that some phosphorylated S6K1 may move back into the nucleus based on the wider distribution of fluorescence lifetimes obtained in the nucleus. We also note that in low to moderate expressing SensOR cells this effect was still apparent, although not so distinct, as lower lifetimes (2.3 ± 0.09 ns) were observed in the cytoplasm with slightly longer lifetimes (2.4 ± 0.07 ns) observed in the nucleus (Supplementary Fig. S9a). In addition, a SensOR construct harbouring a threonine mutation to alanine on amino acid position 389 (SensOR-T389A) was made for validating the wildtype SensOR. The phospho-mutant construct was co-expressed in live HEK293 cells and showed diminished phosphorylation by Western blot analysis. The absence of shorter excited state lifetimes (<2.1 ns) in the cytoplasm in comparison to the wildtype SensOR by FLIM (Supplementary Fig. S9b) suggest a non-phosphorylated form. Together these results identify the localisation of folded SensOR (shorter lifetimes) in the cytoplasm as sites of mTOR mediated phosphorylation in the living cell. However, the observed lifetime of 2.3–2.4 ns indicates a somewhat bent conformation that is neither fully open (2.5 ns) nor fully closed (2.1 ns). The inability for the mutant to be phosphorylated has been further validated by western blot analysis.

**Figure 5:**
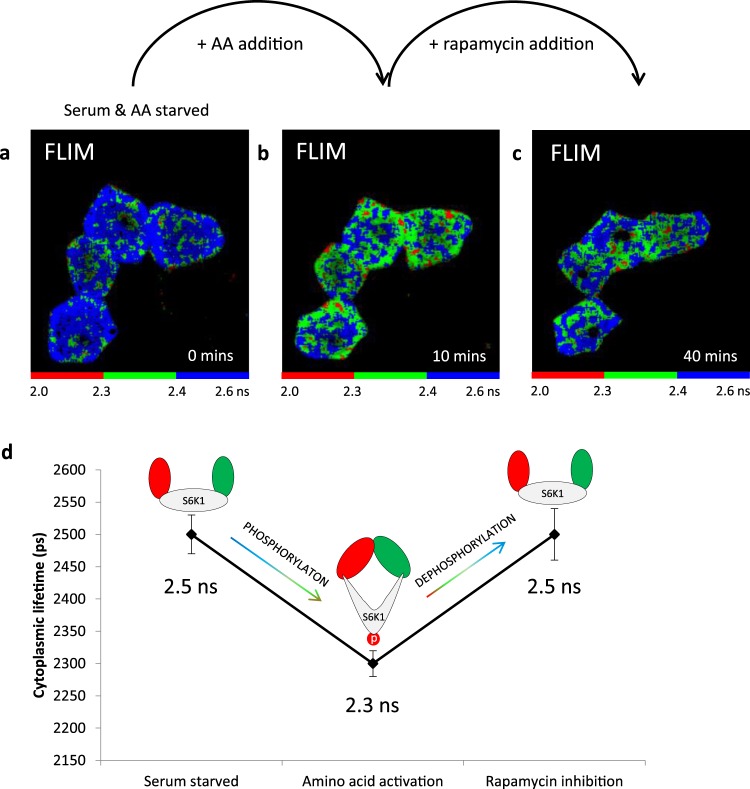
mTORC1 activation and inhibition using SensOR. (**a**) FLIM of serum and amino acid starved HEK293 cells expressing SensOR. (**b**) FLIM at 10 minutes following serine + leucine activation. (**c**) FLIM collected at 40 minutes after subsequent rapamycin treatment of serine + leucine activated cells for 30 minutes. (**d**) Summary of lifetime changes of SensOR with serum starvation, amino acid addition and rapamycin treatment from mean lifetimes and also pixel by pixel analysis of image with increased binning. Opening and closing of sensor also shown via schematics. Data representative of three independent experiments with errors representative of standard deviation, where two experiments were treated with leucine and the third treated with the combination of leucine and serine.

Since the conformational state of S6K1 may be linked to phosphorylation, SensOR was then used to investigate mTOR activity in real-time. Serum and amino acid starvation of HEK293 cells was performed and FLIM of the SensOR resulted in a lifetime of 2.5 ± 0.03 ns in the cytoplasm which upon the addition of amino acids (leucine or serine and leucine) resulted in changes in lifetime to 2.3 ± 0.02 ns in the cytoplasm. Subsequent addition of rapamycin resulted in SensOR revert in lifetime to 2.5 ± 0.04 ns, thus demonstrating the capability of this molecular sensor to explore mTORC1 activity in real time in the live cell (Fig. 5). To confirm a correlation between fluorescent lifetime and S6K1 phosphorylation upon conformational changes, we expressed the SensOR in SF9 insect cells using the baculovirus system, extracted and purified the protein. The fluorescence lifetime of purified SensOR at room temperature was 2.6 ± 0.01 ns, reducing to 2.0 ± 0.08 ns in the presence of ATP strongly suggesting that phosphorylation of the protein is associated with a conformational change in the protein i.e. from an open to closed (or folded) structure. This was further supported by Western blot analysis which showed a 4.7-times increase in SensOR phosphorylation with ATP treatment (Supplementary Fig. S9a).

**Figure 6:**
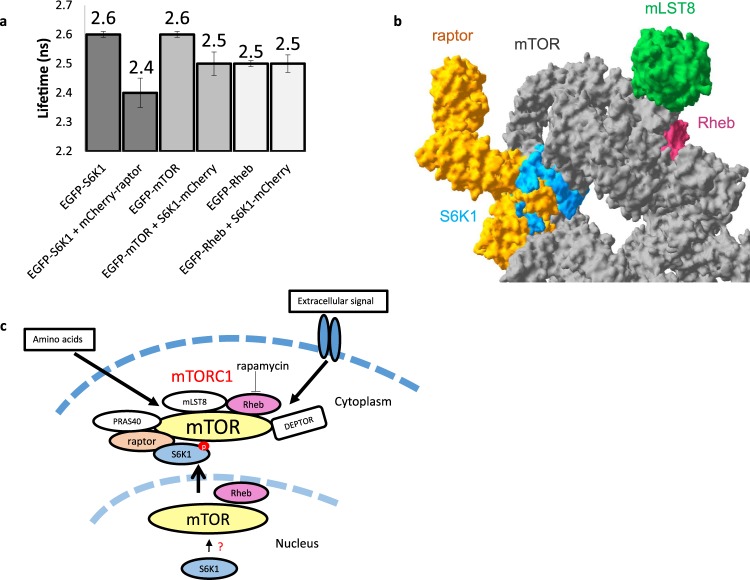
Summary of FRET-FLIM results and revised pathway and mTORC1 structure. (**a**) Summary of FRET-FLIM studies for S6K1-mTORC1 interactions (error bars = SD). (**b**) 3D structural model of S6K1 docked onto the mTORC1 complex made in Swiss PDB Viewer V4.10 software using PDB files: 3a62, 1xts, 5h64. Weak/ transient interaction with mTOR while strong interaction with raptor shown from a monomeric prospective, although the mTOR dimer formation could influence the stoichiometry. (**c**) Schematic of mTORC1 signalling in live cells showing possible S6K1 recruitment onto mTORC1. Modified from Yadav *et al*.^21^.

On the basis of our observations using fluorescent lifetime measurements in live cells and *in vitro*, we conclude that S6K1 exists in a tightly folded state in the cytoplasm associated with phosphorylation and that this involves an interaction with the mTORC1 component raptor (Fig. 6).

## Discussion

The confocal microscopy imaging results show an equally distributed nucleocytoplasmic distribution of S6K1 in live mammalian cells in agreement with fractionation studies^11^ and also clarifying immunofluorescence studies carried out using anti-S6K1 antibodies (STJ31332, St Johns Laboratory, UK) to S6K1 in fixed HEK293 cells^41^. However, the live cell data in this study indicate differences between this study and previous works where cell fractionation work using HEK293 cells^42^ and imaging in fixed HEK293T cells expressing GFP tagged S6K1^13^ showed presence of S6K1 only in the cytoplasm. It is worth noting that S6K1 studies in live plant cells also reported equal nucleocytoplasmic distribution^14^. Whilst recognising these differences in the literature, we believe that our live cell imaging is more suited for the application of soluble and mobile proteins within their natural, physiological and dynamic environment *in situ*, avoiding physical interference that disrupt cellular integrity^43–45^. The current live cell localisation of EGFP-S6K1 provides a more valid insight into the behaviour of the S6K1 protein within a natural context by supporting a nuclear role of S6K1 in processes such as histone phosphorylation^46^ as well as a cytoplasmic role for functions such as cell growth and survival that involves S6K1 in the mitochondria^47^. Here we demonstrate that there may be a dynamic phosphorylation process occurring in the cell cytoplasm whilst the nucleus shows some dephosphorylation as indicated by the FLIM results (above).

A novel aspect of this work revolves around identifying the translocation of full length mammalian S6K1 out of the nucleus when co-expressed with raptor in live HEK293 cells. This translocation may represent the first steps in S6K1 recruitment onto the mTORC1 complex by raptor as fluorescently tagged mTORC1 subunit proteins such as mTOR, raptor and Rheb are predominately localised in the cytoplasm^21,28,48^. This supports the current dogma as several studies have pulled-down S6K1 and raptor together^23,29,31,49^ while full length S6K1 has failed to pull-down with mTOR^50,51^, suggesting that raptor may indeed act as a ‘scaffold’ or stabiliser protein. Further evidence for this notion is supported by the fact that knocking-out raptor inhibits S6K1 phosphorylation^10^. If raptor, which is cytoplasmic, is indeed involved in S6K1 recruitment, which is both cytoplasmic and nuclear, one must begin to question the dynamics of mTORC1 and ask if there is a ‘recruitment’ mechanism involved, driven by a lack of raptor in the nucleus? We discuss this below.

It is interesting to note that both Rheb and raptor interact strongly with mTOR in live cells as demonstrated in previous studies^21^. However, the fact that we observe a 100 ps change in donor lifetime between EGFP-mTOR and S6K1-mCherry (see summary Fig. 6a), considered as a small difference, can be explained by either a fast dynamic interaction which due to FRET-FLIM averaging events over several tens of seconds to acquire an image, may slew the lifetime distribution observed towards the dominate lifetime form or a long-range (>10 nm) interacting distance. Note, that the overall instrument response of our FLIM instrument is around 31 ps. An alternative explanation is that the low level of interaction observed is due to an absence of correct mTORC1 protein levels as high raptor expression, in excess of endogenous raptor, is required to recruit overexpressed S6K1 onto the complex. Similarly, the observation that a threshold level of Rheb is required for dephosphorylation is interesting^18,23,52^.Taking together our results with those previously reported mTOR interactions^21^, we propose a 3D model of the complex using a predicted space-filled representation of an up-to-date snapshot of the assembly of the complex with S6K1 (Fig. 6b). Although a S6K1-raptor-mTOR crystal structure is not yet available, the recent developments of the highly resolved obligate mTOR dimer with raptor structure^53,54^ indicate that the N-terminus of raptor is within close proximity to mTOR. This is consistent with the idea that raptor may bind to S6K1 through the TOS motif and bring S6K1 into the active site of mTOR for phosphorylation. Disrupting the recruitment of S6K1 onto the mTORC1 by raptor, may offer a new opportunity for the design and development of drugs that block mTOR signalling.

Considering the over-expression of Rheb is shown to increase the amount of phospho-S6K1 by 2.3 times, the mechanism by which the increased amount of phosphorylation occurs warrants some thought as we do not observe a direct interaction between Rheb and S6K1. Recently, intricate kinetic and computational modelling of mTORC1 signalling has been developed to inform on the regulation of the complex and also to obtain an insight into the role of Rheb. One such model has found that Rheb may increase S6K1 phosphorylation by increasing mTORC1 substrate binding to raptor^55,56^. This model is further supported by pull-down evidence showing that Rheb overexpression increases 4EBP1 binding to raptor^57^ but we do not observe the necessary translocation of S6K1 with Rheb co-expression.

On the other hand, we find that raptor is a dominant player in the S6K1 translocation process and possibly in stabilising the recruitment of S6K1 onto the mTOR complex. This substrate recruiting role is supported, firstly by our observation that there is an increase in S6K1 phosphorylation upon overexpression of raptor and secondly by the observations that GFP tagged 4EBP1 and YFP tagged PRAS40 inhibit the translocation. These proteins are known to be inhibitors of S6K1 phosphorylation and bind to the same site on the raptor protein as S6K1^26,37,38^. Studies using S6K1 mutants that prevent binding to raptor also show diminished phosphorylation and pull-down interaction^29,31^ which support our S6K1-mutant live cell studies and collectively provide a direct link between translocation and phosphorylation.

Reasons for translocation of S6K1 by raptor need to be examined. It has been shown that mTOR shuttling to the nucleus is required for maximal S6K1 phosphorylation^58,59^. This leads us to believe that mTOR may be partly responsible for the S6K1 translocation effect (first steps of recruitment) as we have previously shown a significant pool of mTOR protein (~40%) in the nucleus of mammalian cells^21^. Expressing S6K1-mTurqouise2, raptor-YFP and a non-functional N-terminal truncated mTOR-mCherry construct resulted in inhibition of the S6K1 translocation effect with a localisation resembling S6K1 alone. This suggests raptor may play a crucial role in the final docking and possible stabilisation of the S6K1 to the complex whilst mTOR may have a preliminary role in recruiting S6K1 although the sequence of events is yet unknown (see summary Fig. 6c).

We now turn to the mTOR phosphorylation process in living cells and to the development of SensOR. In order to investigate the localisation as well as the phosphorylation state of S6K1 in cells, we initially investigated immunofluorescence labelling for phosphorylated S6K1 in fixed HEK293 cells which resulted in the loss of soluble EGFP-S6K1 from the cytoplasm (see Supplementary Fig. S8). Problems in studying GFP tagged mTORC1 in fixed cells as well as extraction of soluble protein by fixation has been previously reported^43,60^. Interestingly we did find that when EGFP-S6K1 was co-expressed with fluorescently tagged raptor in fixed cells labelled for phospho-S6K1 using antibodies we observe similar phospho-EGFP-S6K1 localisation to the cytoplasm as translocated EGFP-S6K1 suggesting that translocation is necessary for phosphorylation and that phosphorylation may occur in the cytoplasm. We note this is contrary to previous reports^12,16^ and the mixed set of data emphasises the need to develop real-time live cell imaging methods able to investigate and report on the localisation of the phosphorylation status of S6K1. To address this requirement, we introduce a novel phospho-S6K1 molecular sensor that exploits a change in the protein conformation upon phosphorylation^17^. Such an approach for 4EBP1^61^ has already been employed. We used GFP and mCherry as a FRET pair and attached them to either side of S6K1 making the arrangement able to track a change in conformation that perturbs the distance between the two ends which is observed by a structural change that brings the labelled chromophores closer, resulting in a lessening of the lifetime upon activation and an increasing of the lifetime upon inhibition. Steady state FRET is unable to determine very small structural changes stressing the advantages of time-resolved measurements. Some cells, particularly highly expressing cells, showed translocation of the biosensor even without raptor co-expression, although the amount of untagged raptor in such cells is unknown. The cause of this effect is unknown; one possible explanation is it may be due to enhanced S6K1 shuttling of some highly expressing cells. Nevertheless, the biosensor data shows distinct variations within the cytoplasm with differences in FRET efficiencies; perhaps indicative of mTOR sub-cellular regional activity which unfortunately cannot be determined using diffraction limited confocal based FRET-FLIM microscopy. Overall, the nuclear localisation of the SensOR shows longer lifetimes than its cytoplasmic localisation. This is indicative of majority of the S6K1 phosphorylation occurring in the cytoplasm. We studied the conformational changes of the SensOR *in-vitro* by using a purified recombinant preparation of the protein produced in insect cells. The reduction of lifetime from 2.6 ns to 2.0 ns in the presence of ATP supports a conformational change upon phosphorylation, consistent with our hypothesis that phosphorylated-S6K1 is in a closed state and mainly in the cell cytoplasm.

Thus, in this live cell ‘test tube’ (for the over expressed system), studies together with the SensOR, we are able to assess the subcellular phosphorylation state of mTORC1. The role of the complex proteins, Rheb, raptor, mTOR and S6K1 can now be investigated in real time. For example, the need to increase the level of Rheb to allow rapamycin to function as well as the modulation of raptor levels to effect phosphorylation. The evidence presented here seeks to resolve the current conundrum in the literature whereby the concentrations of rapamycin required for mTOR inhibition varied significantly between cells as well as from lab to lab. Here our data indicates that the level of Rheb within the cells used needs to be regulated. Whilst we have focused on HEK293 cells, other cell lines can be investigating regarding Rheb effects on rapamycin function. This work paves the way for high throughput drug discovery using the SensOR as an effective S6K1 phosphorylation read-out.

## Conclusion

This study has determined the distribution of S6K1 as well as its phosphorylated state in live mammalian cells together with the observation of novel live cell recruitment (translocation). Our data suggests that although raptor is a major requirement, it is not the only recruiting protein of S6K1 onto the mTORC1 in the first instance but may well have a role in cementing the complex together given raptor’s role as a scaffold protein. Using FRET-FLIM technology to monitor direct protein interactions we observed direct interaction between S6K1 and raptor while a lack of interaction was found with mTOR or Rheb. Having placed S6K1 onto the mTOR complex, we observed dynamic S6K1 protein structural changes during phosphorylation using a new mTORC1 molecular sensor (SensOR). The work provides insights and tools for studies seeking to develop drugs that target the mTOR substrate phosphorylation pathway for the treatment of cancer, type II diabetes and age related disease. Our findings suggest that such efforts should focus on inhibitors that function within the cytoplasm and specifically target the interaction of S6K1 with raptor.

## Methods

### Materials and cell culture

cDNA for S6K1 and 4EBP1-GFPSpark constructs were obtained from Sino Biological (China); mCherry-raptor, YFP-mTOR, YFP-PRAS40 and FLAG-mTOR from Addgene (USA). EGFP-mTOR, mDsRed-Rheb, EGFP-Rheb and mDsRed-raptor constructs were cloned previously^21^. Miniprep and Maxiprep kits purchased from Qiagen (Germany). Rapamycin, L-leucine, L-serine and Ponceau S stain were purchased from Sigma-Aldrich (USA).

HEK293 cell line was purchased from ATCC^®^ (USA), tested for mycoplasma contamination, and HEK293F cells were provided by Evotec (UK) Ltd. Cell culture dishes (35 mm × 20 mm) were bought from MatTek (USA).

HEK293 and HEK293T cells were cultured in Minimum Essential Media (MEM) supplemented with 10% Foetal Bovine Serum (FBS), 2 mM L-glutamine and 1% Penicillin-Streptomycin (P/S) for HEK293. HEK293F cells were grown in serum-free media (Gibco/Life Technologies, UK). All culture reagents were from Life Technologies. Cells were incubated at 37 °C with 5% CO_2_ humidified air in T75 culture flasks (Thermo Fisher Scientific, UK) or in 50 mL tubes (Corning^®^). SF9 cells were cultured in SF 900 III media supplemented with 1% P/S at 26 °C.

### Construction of EGFP-S6K1, EGFP-ΔTOS-S6K1, S6K1-mTurq2, S6K1-mCherry, ΔNmTOR-mCherry, raptor-YFP and mCherry-S6K1-EGFP plasmid constructs

EGFP-S6K1 plasmid construct was made by infusion cloning full length S6K1 cDNA from S6K1-GFPSpark (Sino Biological) into an pOPINN-EGFP (Enhanced Green Fluorescent Protein) vector provided by the Oxford Protein Production Facility (OPPF, UK) using the primers in Table 1, read from 5′ → 3′.

**Table 1:** Primers, forward and reverse for EGFP-S6K1.

EGFP-S6K1 Fwd	AAGTTCTGTTTCAGGGCCCGAGGCGACGAAGGAGGCGGG
EGFP-S6K1 Rev	ATGGTCTAGAAAGCTTTATAGATTCATACGCAGGTGCTCTG

Using the QuickChange Lightening Site-Directed Mutagenesis Kit (Agilent), the TOS motif of EGFP-S6K1 was mutated to GFP-F28A-S6K1 using the primers in Table 2.

**Table 2:** Primers, forward and reverse for EGFP- F28A-S6K1.

EGFP-F28A-S6K1 Fwd	AGGACATGGCAGGAGTGGCTGACATAGACCTGGACC
EGFP-F28A-S6K1 Rev	GGTCCAGGTCTATGTCAGCCACTCCTGCCATGTCCT

S6K1-mCherry and S6K1-mTurqouise2 constructs was cloned in a similar manner into a pOPINE-3C-mCherry/mTurq2 vector, also provided by the OPPF using the primers in Table 3, read from 5′ → 3′.

**Table 3:** Primers, forward and reverse for S6K1-mCherry and S6K1-mTurq2.

S6K1-mCherry/ mTurq2 Fwd	AGGAGATATACCATGAGGCGACGAAGGAGGCGG
S6K1-mCherry/ mTurq2 Rev	CAGAACTTCCAGTTTTAGATTCATACGCAGGTGCTCTG

Truncated mTOR (ΔmTOR)-mCherry was constructed by infusion cloning full-length mTOR ORF from EGFP-mTOR into the pOPINE-3C-mCherry vector using the primers in Table 4, read from 5′ → 3′. Sequencing revealed failure of full length ORF infusion but instead a truncated ORF, perhaps due to internal repeats or sequence similarity with primers.

**Table 4:** Primers, forward and reverse for ΔmTOR-mCherry.

ΔmTOR-mCherry Fwd	AGGAGATATACCATGCTTGGAACCGGACCTGCC
ΔmTOR -mCherry Rev	CAGAACTTCCAGTTTCCAGAAAGGGCACCAGCC

raptor-YFP was cloned by infusion cloning full length raptor ORF from mDsRed-raptor into the pOPINE-3C-YFP vector, using the primers in Table 5, read from 5′ → 3′.

**Table 5:** Primers, forward and reverse for raptor-YFP.

raptor-YFP Fwd	AGGAGATATACCATGGAGTCCGAAATGCTGCAATCG
raptor-YFP Rev	CAGAACTTCCAGTTTTCTGACACGCTTCTCCACCG

mCherry-S6K1-EGFP (SensOR) was constructed by three-way fusion of mCherry cDNA to full length S6K1 cDNA with the pOPINE-3C-EGFP (Enhanced Green Fluorescent Protein) vector as previously described^62^, using the primers in Table 6, read from 5′ → 3′:

**Table 6:** Primers, forward and reverse for mCherry-S6K1-EGFP.

mCherry-S6K1 Fwd	GACGAGCTGTACAAGATGAGGCGACGAAGGAGGCG
mCherry-S6K1 Rev	CCTTCGTCGCCTCATCTTGTACAGCTCGTCCATGC
mCherry-S6K1-EGFP Fwd	AGGAGATATACCATGGCCATCATCAAGGAGTTCATG
mCherry-S6K1-EGFP Rev	CAGAACTTCCAGTTTTAGATTCATACGCAGGTGCTC

mCherry-S6K1-EGFP (SensOR) for protein purification was recloned into the OPPF (pOPINEneo-3C-2STREPStop). Primers in Table 7, read from 5′ → 3′:

**Table 7:** Primers, forward and reverse for mCherry-S6K1-EGFP STREP.

mCherry-S6K1 Fwd	AGGAGATATACCATGGCCATCATCAAGGAGTTCATG
mCherry-S6K1 Rev	CACTAGATTCATACGCAGGTGCTCTGGCCGTTTGGAG
mCherry-S6K1-EGFP Fwd	CGTATGAATCTAGTGAGCAAGGGCGAGGAGCTGTTCACCGGG
mCherry-S6K1-EGFP Rev	CAGAACTTCCAGTTTCTTGTACAGCTCGTCCATGCCGA

Plasmid constructs were verified by reverse PCR screens and further validated by Sanger sequencing using T7F primers and appropriate reverse primers at Source Bioscience (UK).

### Expression and purification of mCherry-S6K1-EGFP-STREP

Expression test and protein production in insect (SF9) and mammalian cells (HEK293T) were performed as previously described^63^. Briefly, SF9 cells were plated and then co-transfected using GenJuice (Novagen) with the mCherry-S6K1-EGFP-STREP and a linearised *Autographa californica* bacmid carrying genetic modifications to increase and stabilise protein expression^64^. Protein expression was assessed by fluorescence after transient expression for mammalian cells (HEK293T) usng FuGENE HD (Promega) or after infection with P1 for SF9 cells and the insect cells were selected as the host for large-scale production using P2 virus. After 72 h, cells are collected (15 min, 6000 g, 4 C) and frozen prior to cell lysis (50 mM Hepes, 150 mM NaCl, 3 mM DTT, 0.1% (w/v) CHAPS, inhibitors of proteases cocktail and 25 U/ml benzonase) and centrifugation (13000 g, 30 min, 4 C). Supernatant was then filtered prior to affinity purification using StrepTrap column followed immediately by gel filtration using a Superdex 200 10/300 on AKTA express using manufacturer’ standard protocols (GE Healthcare).

### Cell transfection

HEK293 were seeded for 24 hours at a density of 1 × 10^5^ or 1.5 × 10^5^ cells per mL on uncoated 35 mm number 1.5 glass bottom dishes (MatTek) or seeded and transfected straightway in 50 mL tubes. Cells were transfected with 1 μg of plasmid DNA using FuGENE HD (Promega, UK) transfection reagent or Polyethylenimine (PEI) Max.

### Pull-down S6K1-mTORC1 interactions

Following 72 hours of HEK293F (suspension cells) transfection, 20 mL of 1 × 10^6^ cells per mL were centrifuged and the cell pellet was lysed in 1 mL of lysis buffer (50 mM HEPES, pH 7.4, 150 mM NaCL, 3 mM DTT, 0.4% CHAPS and protease inhibitors) by ultrasonication (30 sec on/off cycle) for 4 minutes on ice. Lysate was spun-down by centrifugation for 45 minutes and soluble fraction was loaded onto a 10 µl Ni-NTA resin PhyTip using a PhyNexus robot at 4 °C, washed (wash buffer: lysis buffer + 20 mM imidazole) and eluted (elution buffer: lysis buffer + 500 mM imidazole) in 40 µl final volume. Eluted proteins were separated on midi-gels (Bio-Rad, UK) for coomassie staining and Western blotting. Briefly, for Western blot analysis of purified complexes, gels were transferred and blocked in 5% milk blocking buffer for 1 hour and labelled for proteins of interest using 1:1000 dilutions of anti-His (Bio-Rad), anti-Rheb (Santa Cruz), anti-FLAG (Sigma Aldrich) and anti-raptor (Cell Signalling) antibodies  overnight. Blots were washed three times in PBST (1X) before incubation with relevant secondary conjugated antibodies (anti-AP) using 0.3:1000 dilutions. Blots were washed three times in PBST (1X) and once in deionised water before development with Enhanced Chemi-Luminescence (ECL) or 5-Bromo-4-Chloro-3-Indolyl-Phosphate with Nitro Blue Tetrazolium (BCIP/NBT) substrate solution before imaging.

### Fixing and immunofluorescence labelling cells for phospho-S6K1

Following 48 hours transfection and mock transfection, HEK293 cells were fixed with 4% paraformaldehyde in 1X Phosphate Buffered Saline (PBS) (Life Technologies) for 15 minutes or fixed in ice-cold methanol for 20 minutes at −20 °C and washed three times in cold PBS. Cells were permeabilised with 0.3% Triton X-100/PBS/0.1% Bovine Serum Albumin (BSA) solution (Sigma-Aldrich) and washed three times with PBS. Fixed cells were blocked with 5% BSA in PBS to reduce non-specific binding and incubated with 1:200 anti-Phospho-S6K1 (T389/T412) primary antibody (STJ91045) or anti-S6K1 (STJ31332, St Johns Laboratory, UK) at room temperature for 1 hour, washed three times and incubated with 1:500 Alexa Fluor 405 or 555 (Thermo Fisher Scientific) secondary antibody for 1 hour in dark. Cells were washed twice and maintained in PBS before imaging.

### Immunoblot analysis of phospho-S6K1 phosphorylation

Following 48 hours transfection and mock transfection, HEK293 cells were washed once with PBS (1X) and detached using 1X trypsin for 5 minutes. Cell pellets were obtained by centrifugation, and suspended in 125 µL of Cellytic M (Sigma-Aldrich) lysis buffer containing 1:1000 dilution of protease inhibitor cocktail (Thermo Fisher Scientific) with added sodium fluoride (NaF) and sodium orthovanadate (Na_3_VO_4_) (Sigma-Aldrich). Total protein was quantified by Bradford reagent (Sigma-Aldrich) and equal protein was loaded into each well of a NuPAGE Novex 10% Bis-Tris protein gel and ran with MES (1X) buffer for 35 minutes at 200 V, from Life Technologies. Gels were transferred to PVDF membrane and blotted with 5% milk for 1 hour, followed by overnight incubation with phospho-S6K1 (Thr389) primary antibody (Cell Signalling) at 4 °C. The PVDF membrane was washed three times with 1X Tris buffered saline with tween (TBST) and blotted with HRP-linked secondary antibody (Cell Signalling) for 1 hour and washed with TBST three times. ECL (Thermo Fisher Scientific) was added for 5 minutes before chemiluminescent imaging. The blot was stripped using Restore Western Blot Stripping Buffer (Thermo Fisher Scientific) for 15 minutes and re-blotted for vinculin or same gel stained with Ponceau S stain solution. All gels were imaged using Bio-Rad Chemidoc™MP Imaging system. Settings for western blot image acquisition were set to ‘auto’ in order to obtain best contrast and gain. Western blot antibody to RPS6 is a kind gift from Dr Ken Raj from Public Health England, Harwell, UK and used as 1:1000 dilution of phospho-S6 Ribosomal Protein (Ser235/236) Antibody (#2211) from Cell Signalling.

### S6K1 FRET bio-sensor cell starvation, activation and inhibition studies

Following 48 hours transfection, cells were washed once with pre-warmed PBS (1X) and incubated in pre-warmed serum-free medium overnight. Cells were amino acid starved for 1–2 hour prior to imaging by washing the cells once with PBS and incubating them in pre-warmed Dulbecco’s phosphate-buffered saline (DPBS) containing magnesium and calcium. Leucine (1 mM) and serine (500 mM) were made in full serum media and added dropwise to initiate activation. Rapamycin (250 nM) was made in full serum media and also added in a dropwise manner to initiate inhibition.

### Confocal and FLIM setup

Confocal images were taken using an inverted Nikon TE2000-U or Ti-E microscope attached to a Nikon C1 or C2 scanning unit with a GFP (488 nm excitation) or mDsRed/mCherry/Alexa 555 (543 or 561 nm excitation) and mTurqouise2/Alexa405 (405 nm excitation) filter set or using a Leica TCS SP8X confocal microscope using pre-set GFP, mCherry software settings and filters. For multiphoton FLIM acquisition, the settings and setup used has been described previously^21,65^. Briefly, multiphoton excitation using 910 ± 5 nm from a mode-locked titanium sapphire laser (Mira, Coherent Lasers) that generated pulses of 180 femtoseconds at 76 MHz from a 532 nm laser pump source (Verdi, Coherent Lasers) was used. FLIM images were acquired at 256 × 256 pixels compared to previous 128 × 128 pixels and 920 ± 5 nm multiphoton excitation^21^ or using a 488 nm one-photon excitation (NKT supercontinuum laser). Collected fluorescence emission was passed through a bandpass filter (BG39, Comar) or 515/30 (Thorlabs) filter.

## Supplementary information

Supplementary Information

## Data Availability

All raw data files including images will be available via the STFC ePubs hosting site for access by other researchers.
