# Supplementary material for: Direct imaging of the recruitment and phosphorylation of S6K1 in the mTORC1 pathway in living cells
Source: Sci Rep. 2019 Mar 4;9:3408. doi: 10.1038/s41598-019-39410-z (PMC6399282; doi:10.1038/s41598-019-39410-z)
Supplement: Supplementary file 1 — Supplementary Information [file 41598_2019_39410_MOESM1_ESM.pdf]

Direct imaging of the recruitment and phosphorylation of S6K1 in the mTORC1 pathway in living cells

Abdullah R Ahmed[1], Raymond J. Owens[3,4], Christopher D Stubbs[1], Anthony W Parker[1], Richard Hitchman[2], Rahul B Yadav[2], Maud Dumoux[5.3], Chris Hawes[6] and Stanley W Botchway[1*]

[1] Central Laser Facility, Research Complex at Harwell, STFC Rutherford Appleton Laboratory, Harwell Campus, OX11 0FA, UK

[2] Evotec (UK) Ltd, 114 Innovation Drive, Milton Park, Abingdon, Oxfordshire, OX14 4RZ, UK

[3] Protein Production UK, Research Complex at Harwell, Rutherford Appleton Laboratory, Harwell Campus, OX11 0FA, UK

[4] The Wellcome Centre for Human Genetics, Roosevelt Drive, Oxford OX3 7BN, UK

[5] Diamond Light Source, Harwell Campus, OX11 0DE, UK

[6] Oxford Brookes University, Headington Campus, Oxford, OX3 0BP, UK

* Correspondence: stan.botchway@stfc.ac.uk

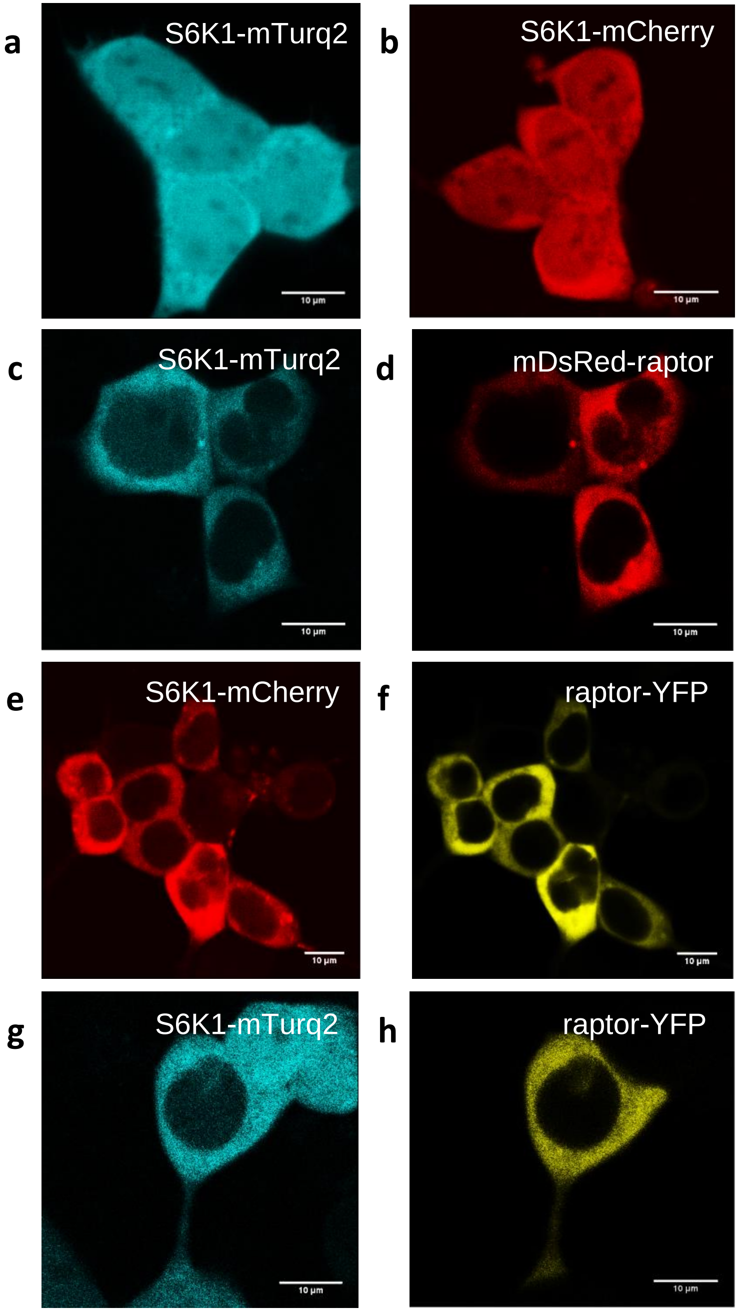

Supplementary Figure S1a: Live cell imaging of S6K1 and raptor tagged GFP variants. **a** & **b** Confocal images of S6K1- mTurquoise2 and S6K1-mCherry in live HEK293 cells. **c-h** Confocal images of S6K1-mTurq2 and S6K1-mCherry with mDsRed-raptor or raptor-YFP co-expression in live HEK293 cells.

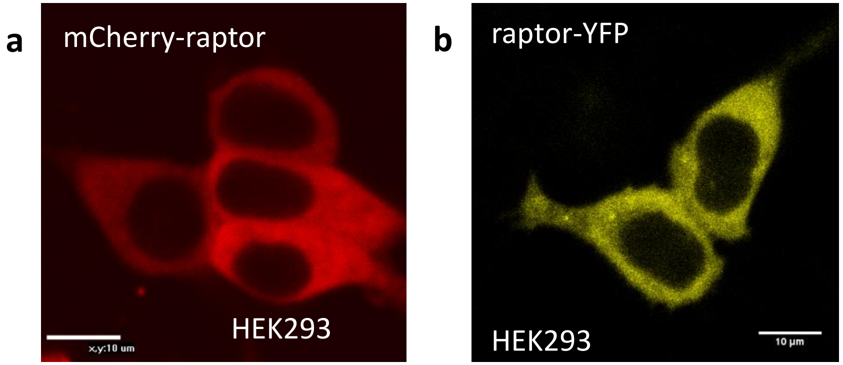

Supplementary Figure S1b: Live cell imaging of raptor tagged GFP variants. Confocal images of **a** mCherry-raptor, **b** raptor-YFP. Scale bar = 10 µm

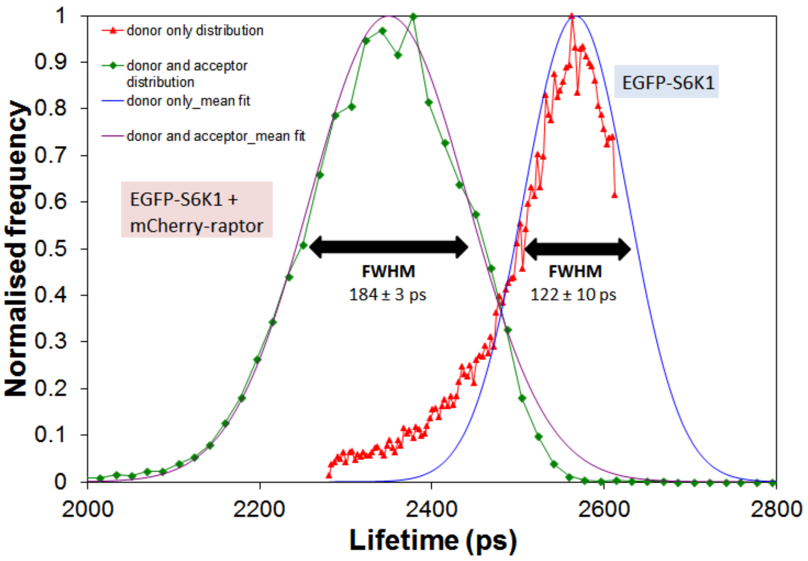

Supplementary Figure S2a: Lifetime distribution of EGFP-S6K1 (donor) with EGFP-S6K1 and mCherry-raptor (acceptor) fitted in MATLAB V.R2013A in conjunction with ezyfit software (V.2.42) to a Gaussian distribution 'a*exp(-((x-x_0)^2)/(2*sigma^2))' where x_0 = mean and 2*sigma is Full Width at Half Maximum (FWHM). Exported lifetime histogram data from Figure 2 was used. Data resolved at FWHM showing good separation.

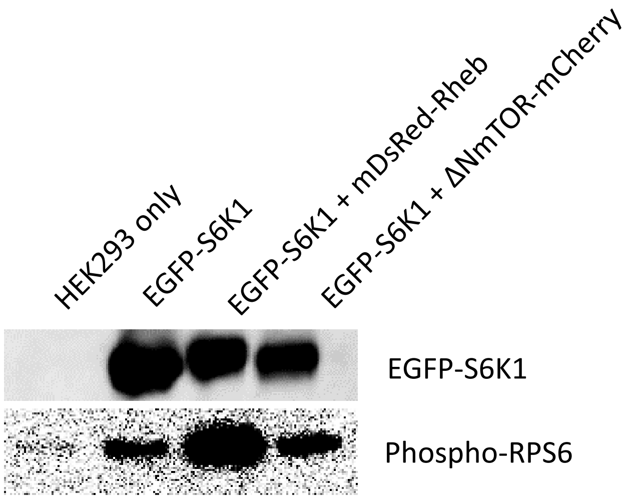

Supplementary Figure S2b: Overexpression of EGFP-S6K1 induces an increase in endogenous phospho-RPS6 by 6 fold, lane 2.Rheb increase phospho-RPS6 too which correlative to S6 activity, lane 3. The truncated mTOR shows an inhibitory effect as EGFP-S6K1 only levels is heightened in the total S6K1 loading. Full-length blots are presented in Supplementary Figure S11

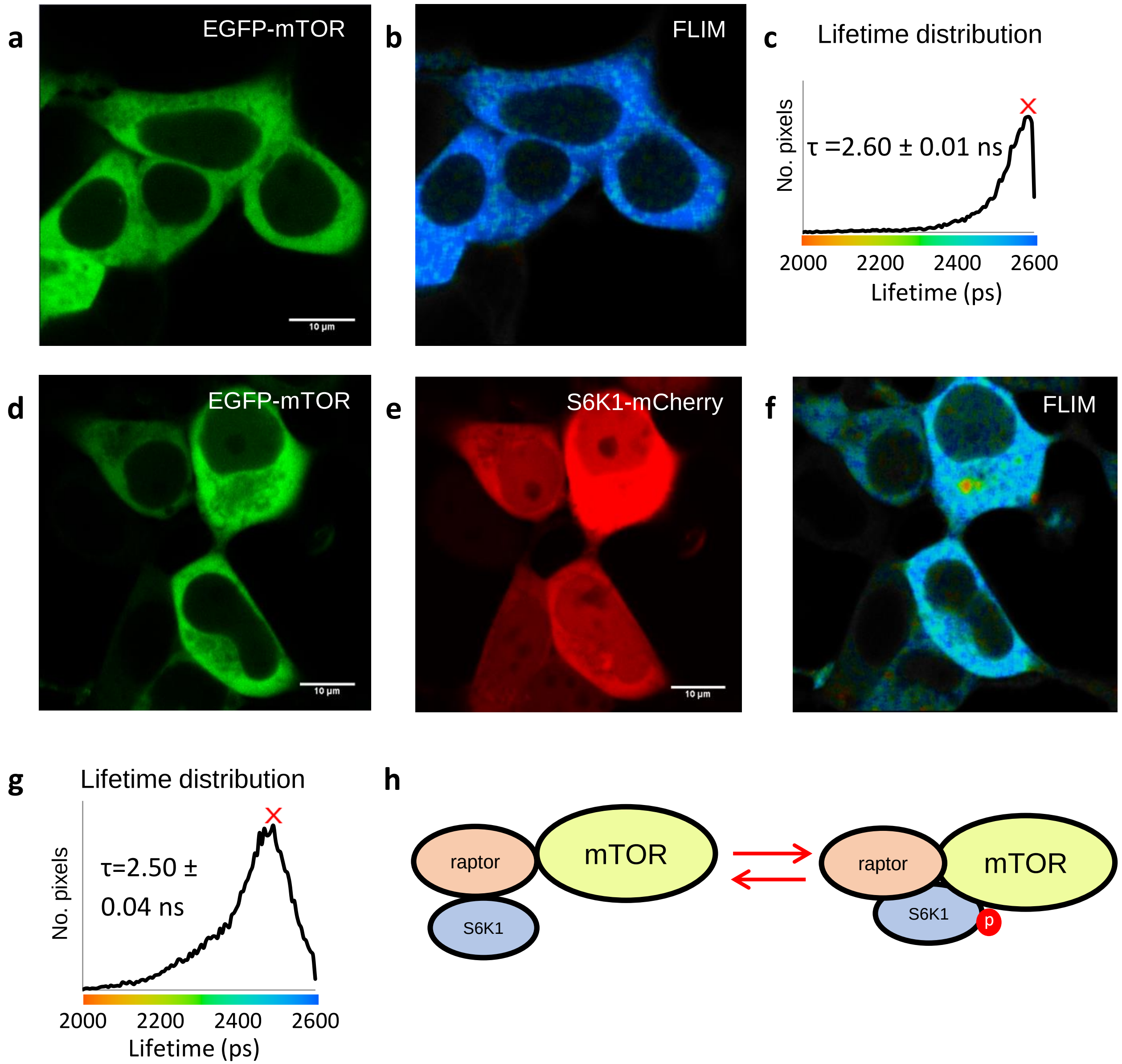

Supplementary Figure S3: Live cell interaction between S6K1 and mTOR using FRET-FLIM in HEK293 cells. **a** confocal image of EGFP-mTOR. **b** & **c** FLIM of EGFP-mTOR and corresponding lifetime distribution histogram with cross showing maximum average lifetime (τ). **d-g** Confocal images of EGFP-mTOR when co-expressed with S6K1-mCherry, FLIM of EGFP-mTOR when co-expressed with S6K1-mCherry and corresponding lifetime distribution histogram shown. **h** Schematic of proposed dynamic interaction where S6K1 may be in a dynamic equilibrium between direct interaction and long distance interaction with mTOR

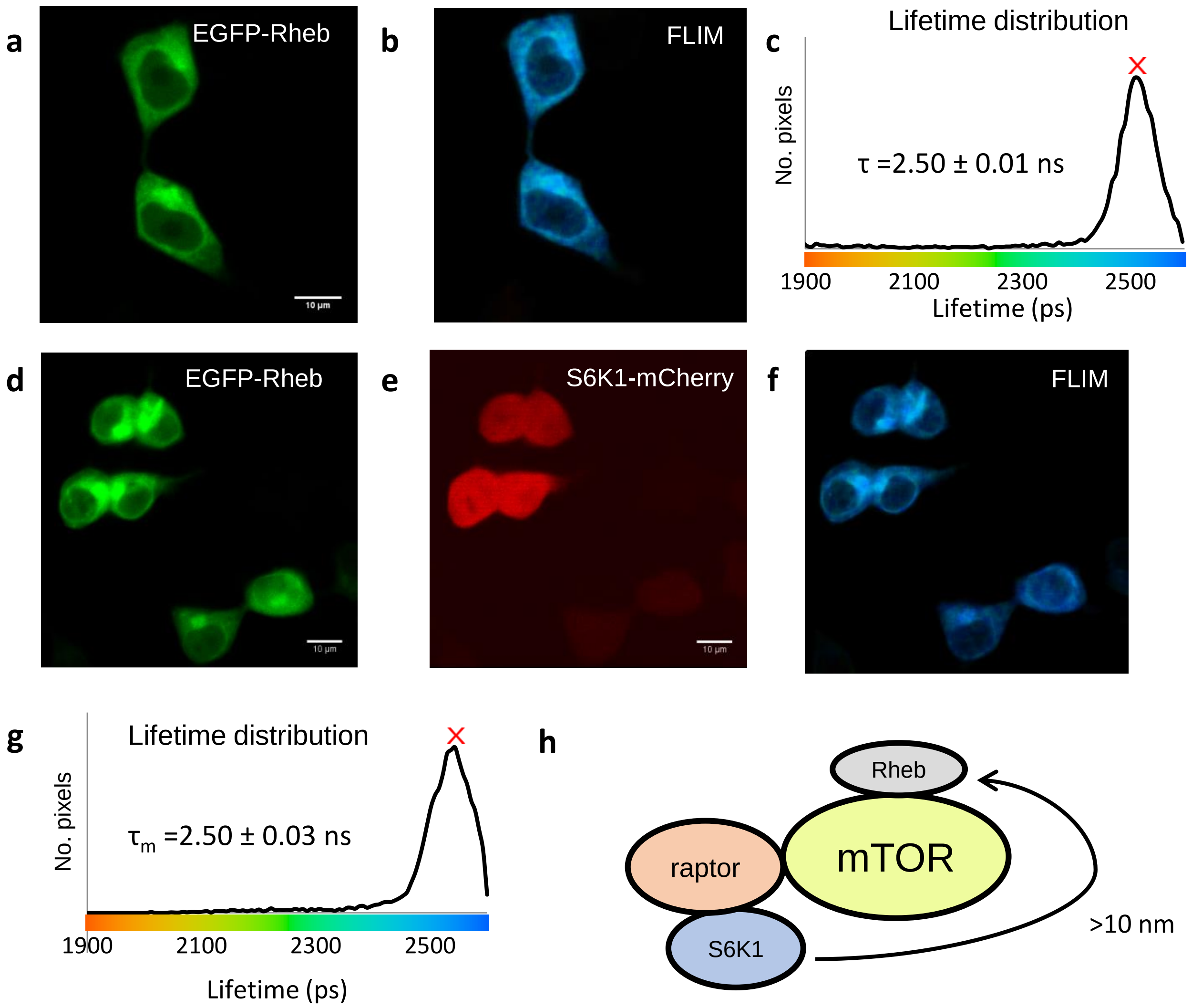

Supplementary Figure S4: Live cell interaction between S6K1 and Rheb using FRET-FLIM in HEK293 cells. **a** confocal image of EGFP-Rheb. **b** & **c** FLIM of EGFP-Rheb and corresponding lifetime distribution histogram with cross showing maximum average lifetime (τ). **d-g** Confocal images of EGFP-Rheb when co-expressed with S6K1-mCherry, FLIM of EGFP-Rheb with S6K1-mCherry and lifetime distribution histogram. **h** Schematic showing proposed long distance interaction where S6K1 is placed onto the complex but away from Rheb

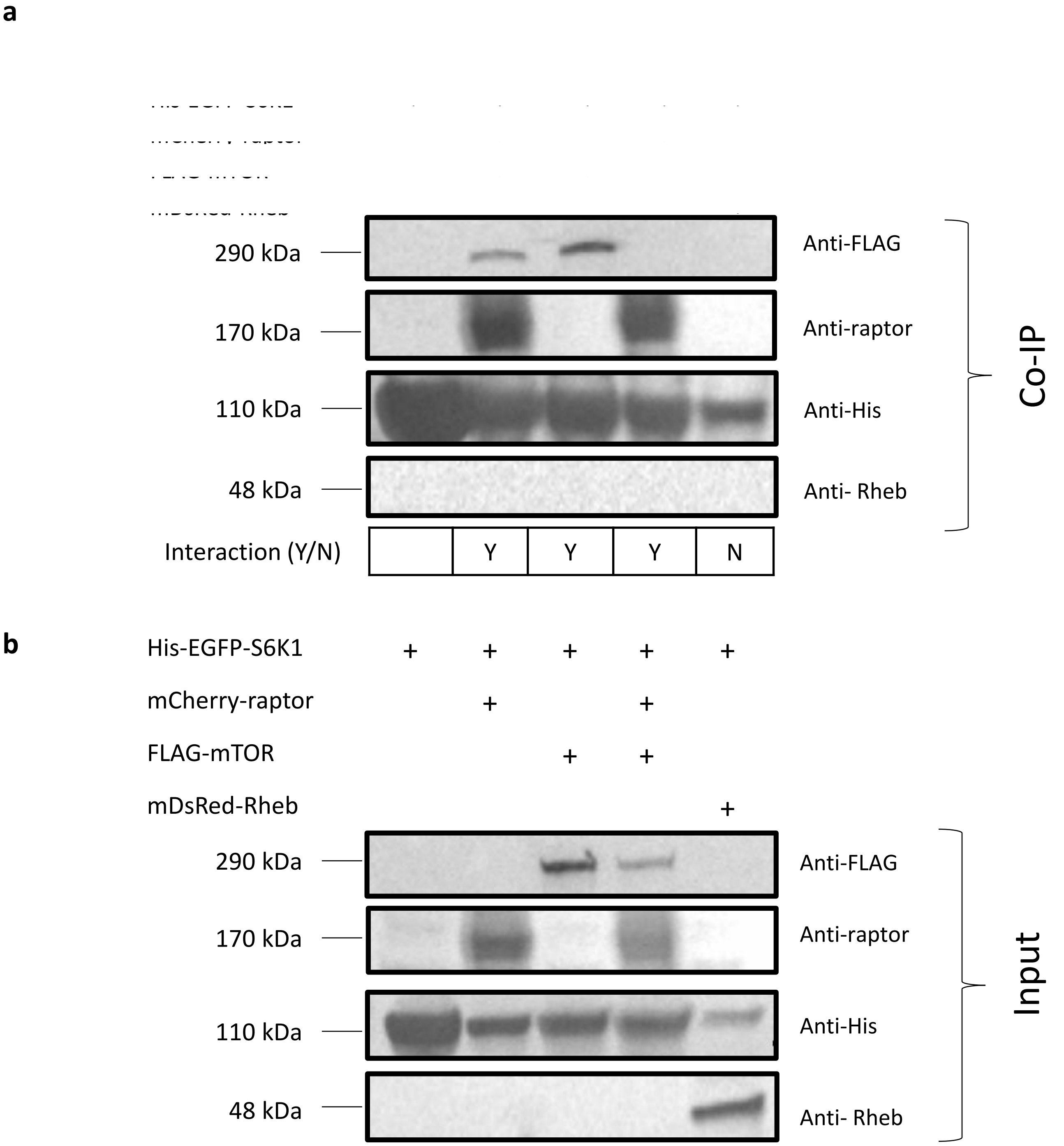

Supplementary Figure S5: S6K1-mTORC1 pulldown to support interactions using Co-IP. **a** Western blots of denatured (by SDS) eluted complexes (from 40 µL of eluted sample) of S6K1 + raptor, S6K1 + mTOR, S6K1 + raptor + mTOR and S6K1 + Rheb are shown using HEK293F cells. Presence of all expected interacting proteins + His-EGFP-S6K1 ("bait" protein) shows interaction in the co-IP blots whilst absence of expected binding protein + presence of His-EGFP-S6K1 ("bait" protein) indicates no interaction. Summary of interactions are shown and assigned (yes/no) Y/N. **b** Lysates are shown (input) of lysed soluble sample before pull-down (from 1 mL of sample). HA-mCherry-raptor was blotted for using anti-raptor antibodies, His-EGFP-S6K1 was blotted for using anti-His antibodies, mDsRed-Rheb was blotted using anti-Rheb antibodies and FLAG-mTOR was blotted for using anti-FLAG antibodies. Full-length gels and blots are presented in Supplementary Figure S12.

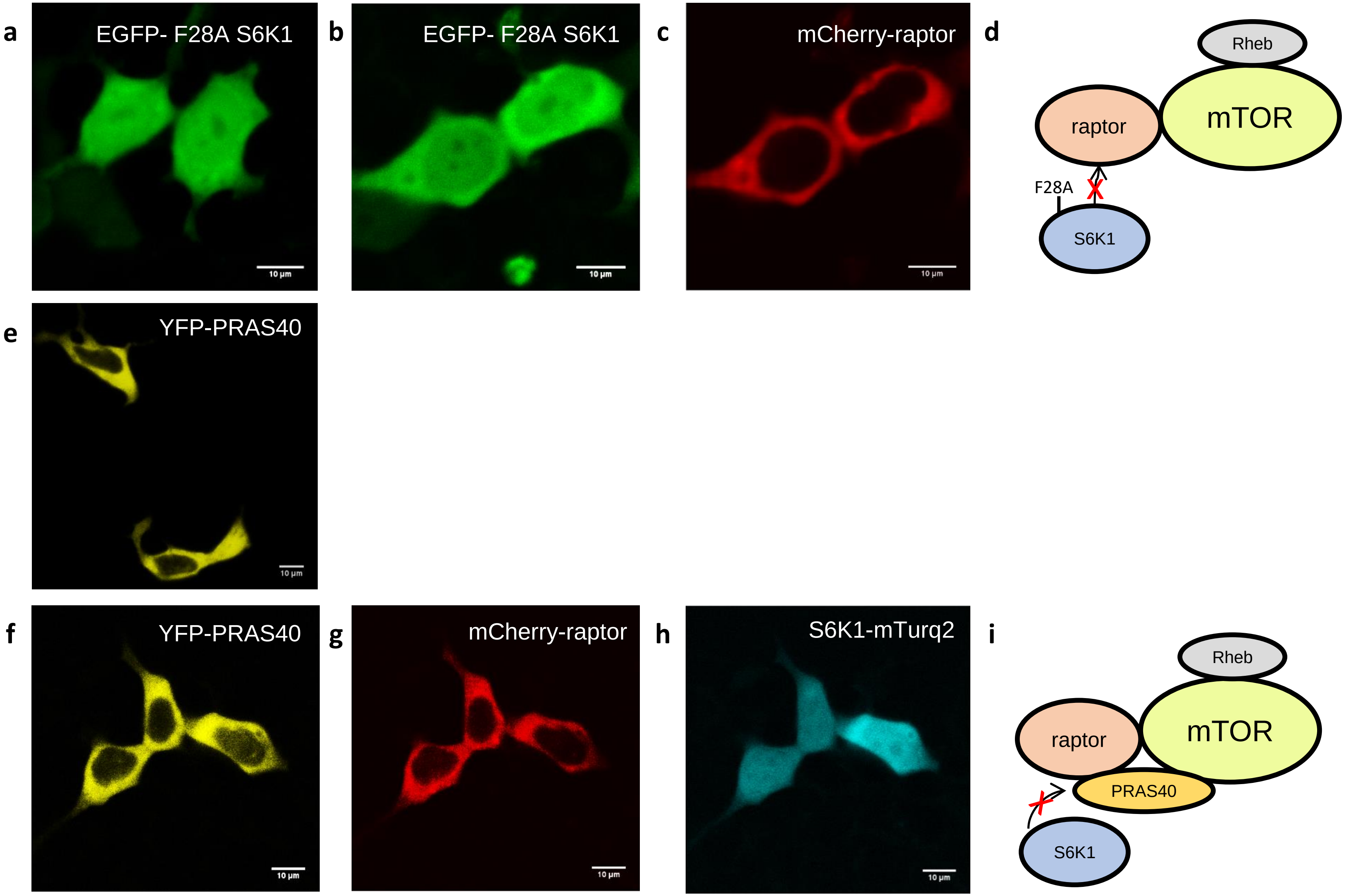

Supplementary Figure S6 : S6K1 TOS-motif mutant & PRAS40 inhibit S6K1 recruitment in live HEK293 cells. **a** Confocal image of EGFP-F28A S6K1 (TOS motif mutant). **b** & **c** Confocal images of EGFP-F28A S6K1 with mCherry-raptor co-expression. **d** Schematic for TOS motif interaction with raptor where mutated S6K1 is unable to bind onto the complex and subsequently can not be phosphorylated. **e** Confocal image of YFP-PRAS40 alone. **f-h** Confocal images of YFP-PRAS40, mCherry-raptor and S6K1-mTurquoise2 triple expression. **i** Schematic of PRAS40 binding to both mTOR and raptor, resulting in an inhibition in S6K1 translocation by competitive binding.

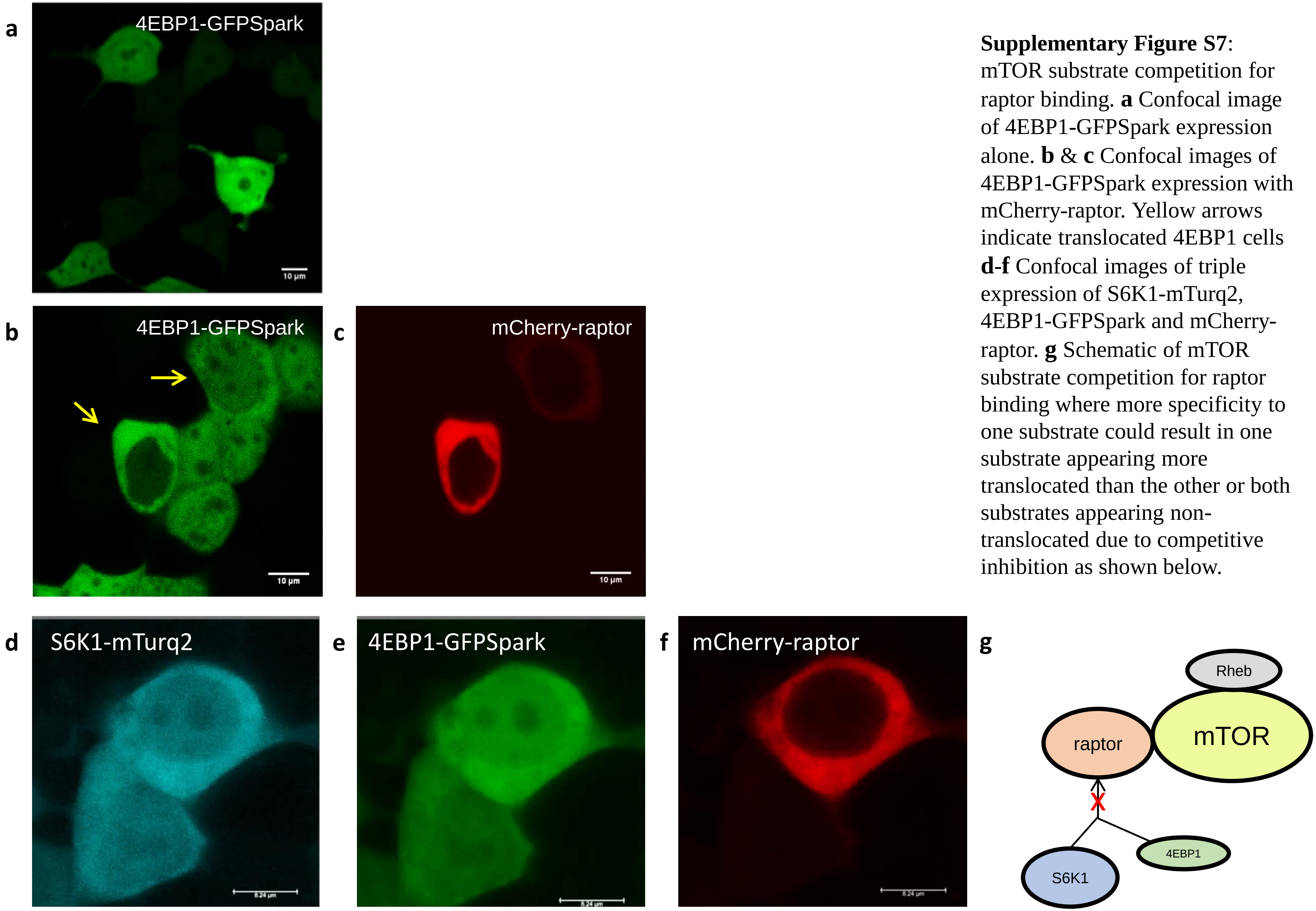

Supplementary Figure S7: mTOR substrate competition for raptor binding. **a** Confocal image of 4EBP1-GFPSpark expression alone. **b** & **c** Confocal images of 4EBP1-GFPSpark expression with mCherry-raptor. Yellow arrows indicate translocated 4EBP1 cells **d-f** Confocal images of triple expression of S6K1-mTurq2, 4EBP1-GFPSpark and mCherry-raptor. **g** Schematic of mTOR substrate competition for raptor binding where more specificity to one substrate could result in one substrate appearing more translocated than the other or both substrates appearing non-translocated due to competitive inhibition as shown below.

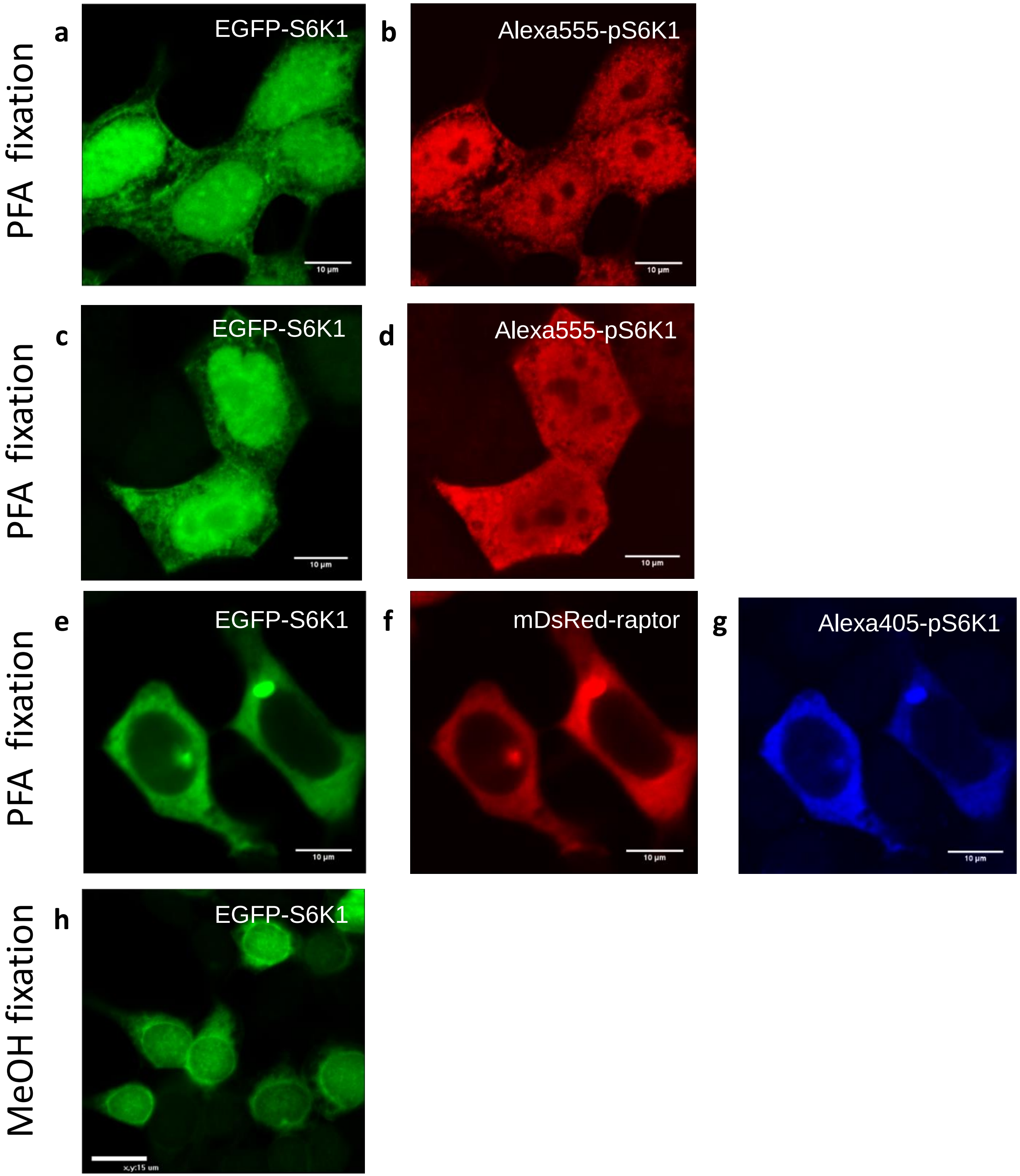

Supplementary Figure S8 : Phospho-S6K1 localisation in fixed HEK293 cells. **a-d** Confocal images of EGFP-S6K1 with labelling for phospho-S6K1 (pS6K1) using Alexa555 antibodies in paraformaldehyde (PFA) fixed cells. **e-g** Confocal images of EGFP-S6K1 with mDsRed-raptor co-expression and conjugated Alexa405 antibody labelling to anti-phospho-S6K1 (pS6K1) in PFA fixed cells. **h** Confocal image of EGFP-S6K1 in methanol fixed cells.

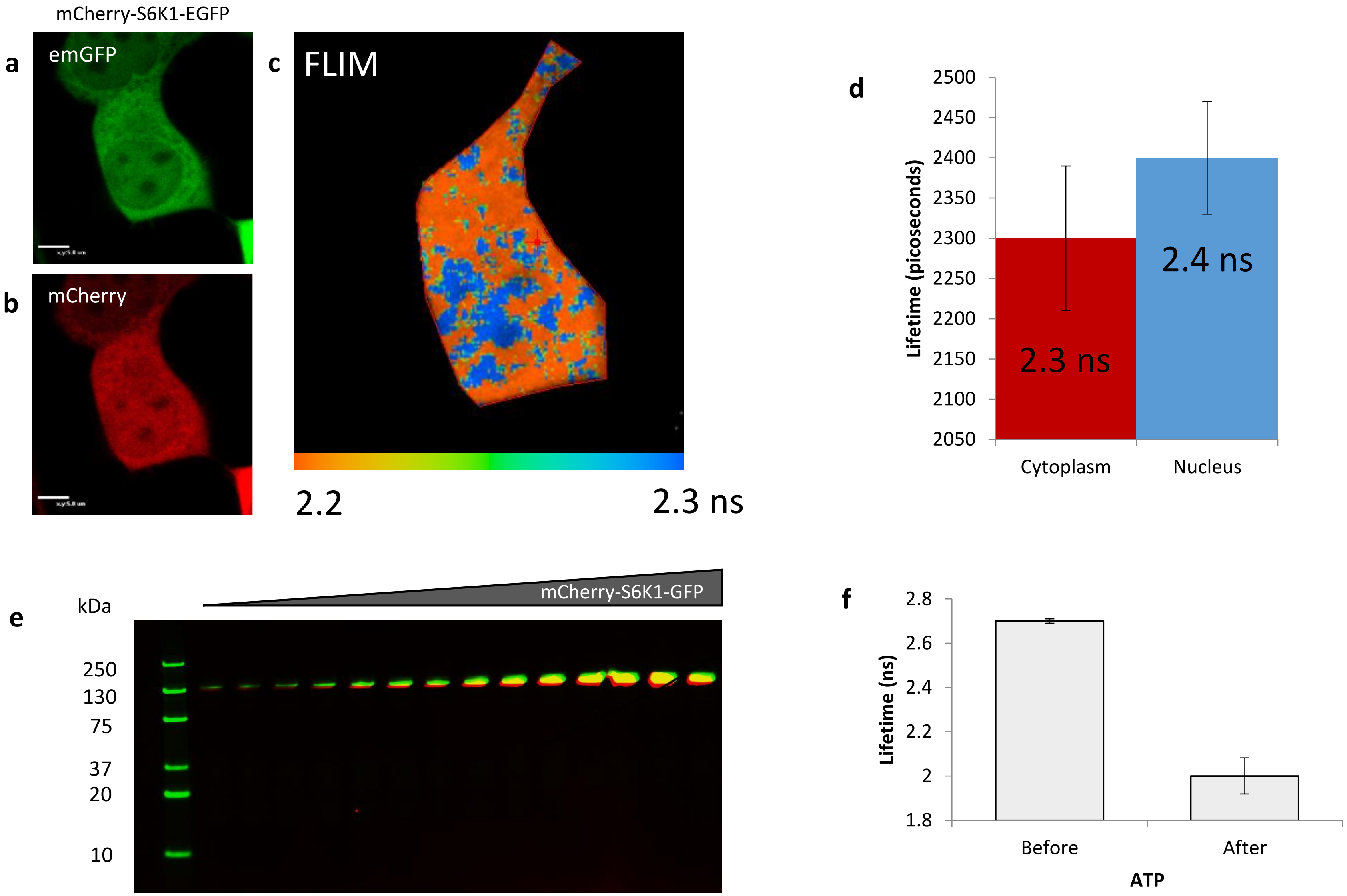

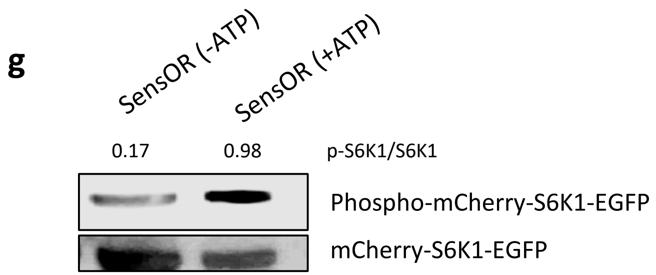

Supplementary Figure S9a : Localisation and cellular lifetimes of SensOR. **a-b** Confocal images showing tandem S6K1 construct. **c** FLIM image of SensOR with lifetime scale. **d** Graph comparing lifetime of SensOR between cytoplasm and nucleus in low to moderate SensOR expressing HEK293 cells. **e** In-gel fluorescence of purified SensOR (final concentration at 3.6 mg). **f** Addition of 2.4 mM ATP in buffer. Reduced lifetime indicates a closed/folded protein structure observed in real time. **g** Western blot analysis of purified SensOR (60 µg) with and without ATP (2.4 mM) probed for S6K1 phosphorylation showing an increase in p-S6K1. Full length gels given in S15. All error bars (SD) are from a minimum of three experiments.

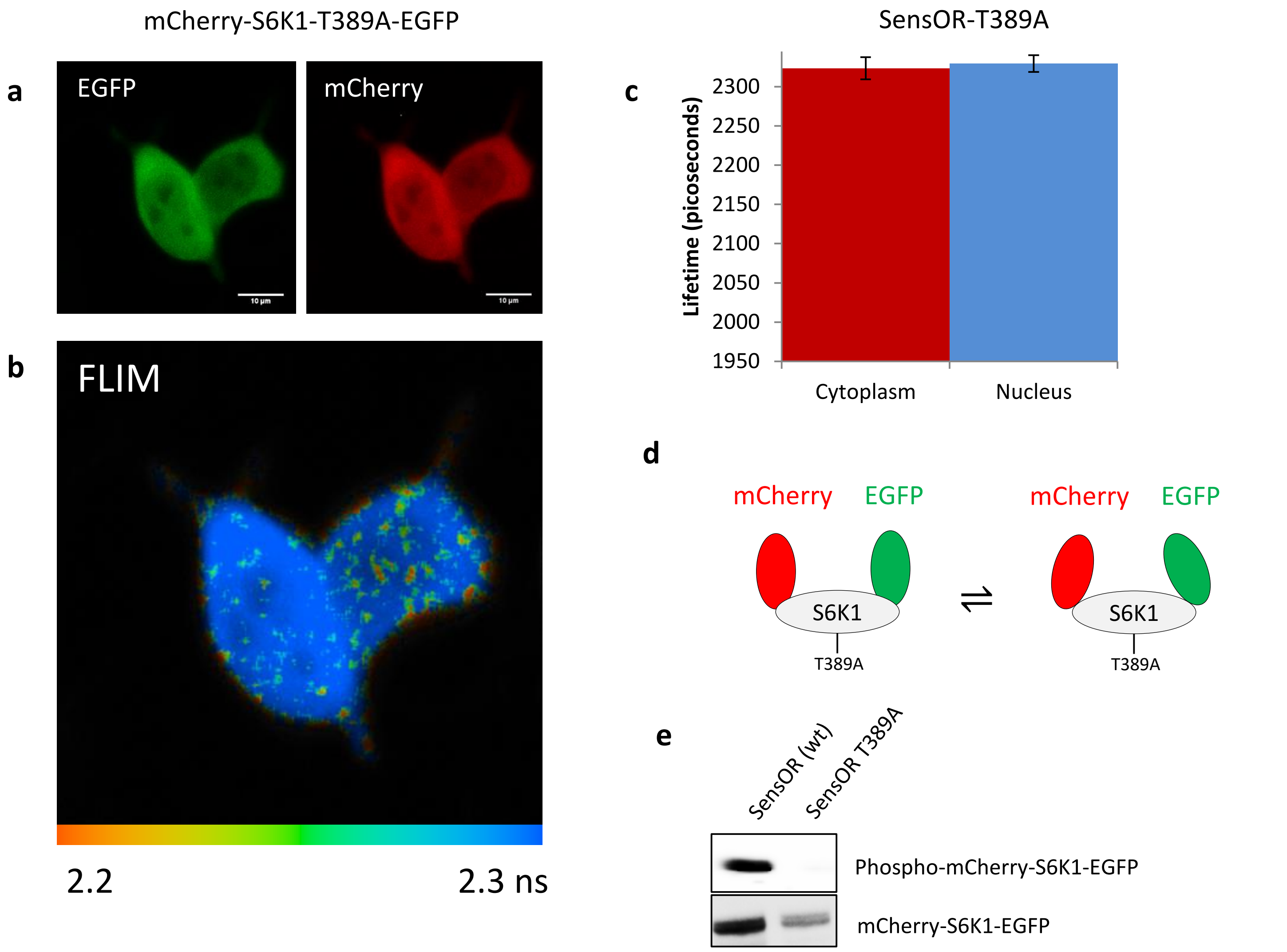

Supplementary Figure S9b: **a** confocal images of mCherry-S6K1-T389A-EGFP (SensOR-T389A) with both green (EGFP) and red (mCherry) channels, **b** FLIM image of mutated SensOR, **c** lifetime scale bar below in nanoseconds (ns) and graph showing cytoplasmic against nuclear lifetime distributions of SensOR, selected by selecting sub-cellular regions and obtaining the mode lifetime in SPCImage V6.0 software. **d** Proposed model of mutant SensOR. **e** Western blot validation of SensOR-T389A for phospho-S6K1 activity. Full length blots provided in supplementary Figure S16.

Full length blots from Figures in the main text and supplementary information above:

Figure 1 are given in S10
Figure S2b are given in S11
Figure S5 are given in S12
Figures 3d&h are given in S13
Figure 4 are given in S14
Figure S9ag are given in S15
Figure S9be are given in S16

Table of antibodies used for the following pull-downs

Anti-Phospho-S6K1 (T389/T412)	St John's Laboratory (UK)	STJ91045
Anti-S6K1	St John's Laboratory (UK)	STJ31332
Anti-Phospho-RPS6 (Ser235/236) (rabbit)	Cell Signalling (UK)	#2211
Anti-Rheb antibody (goat)	Santacruz Biotechnology (UK)	sc-6341
Anti-Vinculin (mouse)	Millipore (UK)	MAB3574
Anti-His (mouse)	Qiagen (UK)	34660
Anti-FLAG (mouse)	Sigma-Aldrich (UK)	F1804
Anti-mTOR (rabbit)	Cell Signalling (UK)	#2983
Anti-raptor (rabbit)	Cell Signalling (UK)	#2280
Anti-rabbit Alexa Fluor 405/ 555	Abcam (UK)	ab175649/ ab150074
Anti-rabbit HRP	Cell Signalling (UK)	#7074
Anti-mouse AP	Promega (UK)	S372B
Anti-rabbit AP	Promega (UK)	S3731
Anti-goat AP	Abcam (UK)	ab6742-1

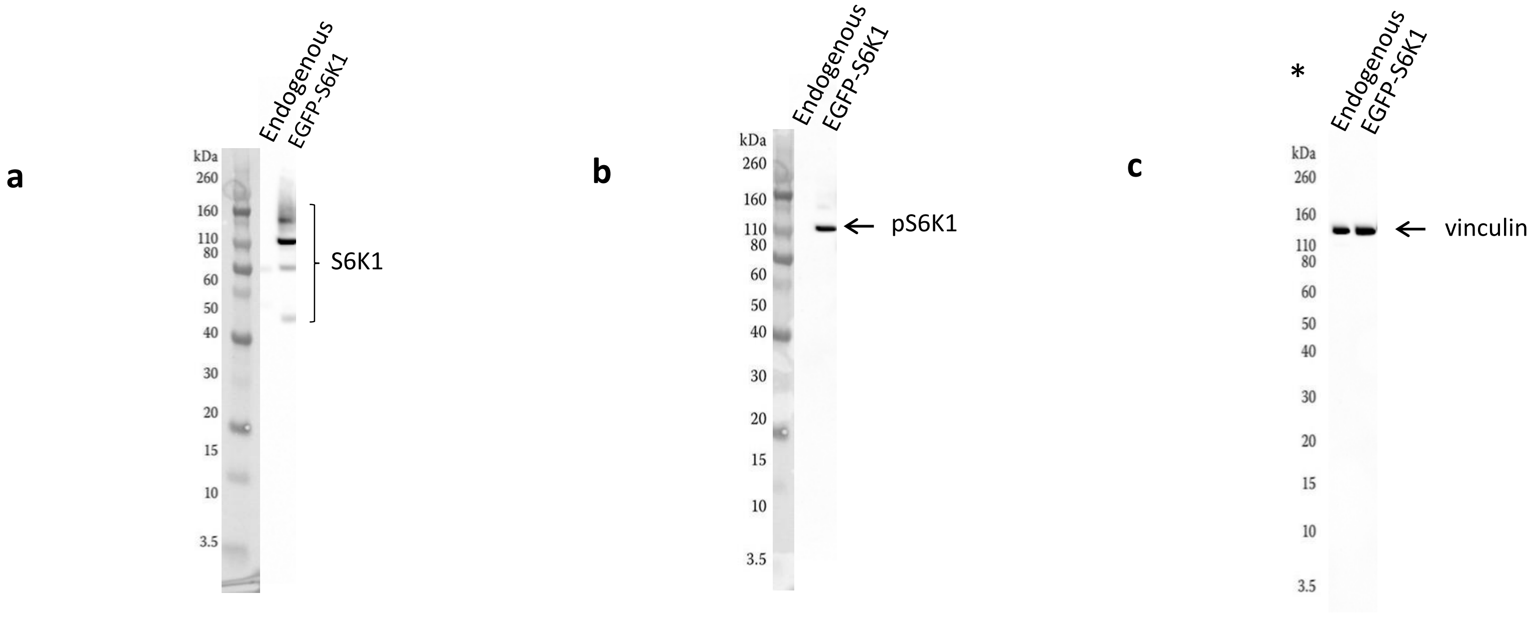

Supplementary Figure S10 – Full lengths gels for Figure 1b.**a** WB for S6K1 using anti-S6K1. **b** WB for p-S6K1 using anti-phospho S6K1 antibody. **c** WB for vinculin using anti-vinculin antibody. Unavailable respective colorimetric marker images are indicated (*)

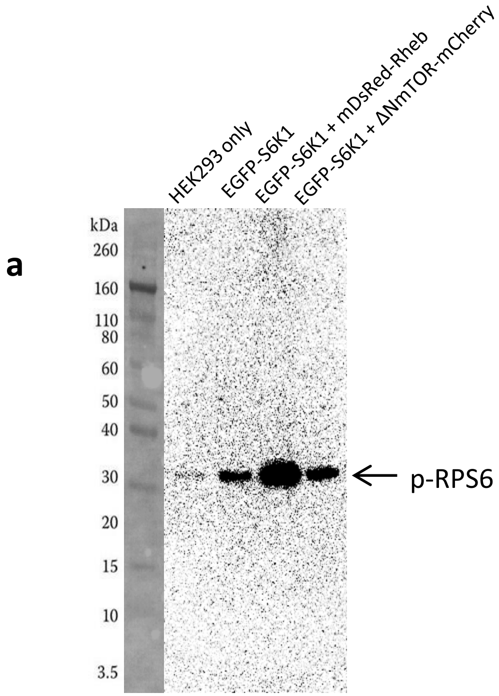

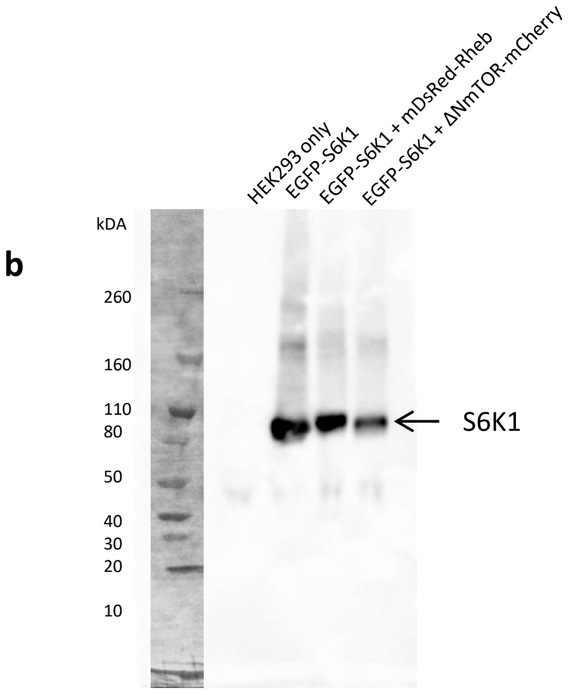

Supplementary Figure S11 – Full lengths gels for Supplementary S2b. **a** WB for p-RPS6 using anti-phospho RPS6 antibody. **b** WB for S6K1 using anti-S6K1 antibody

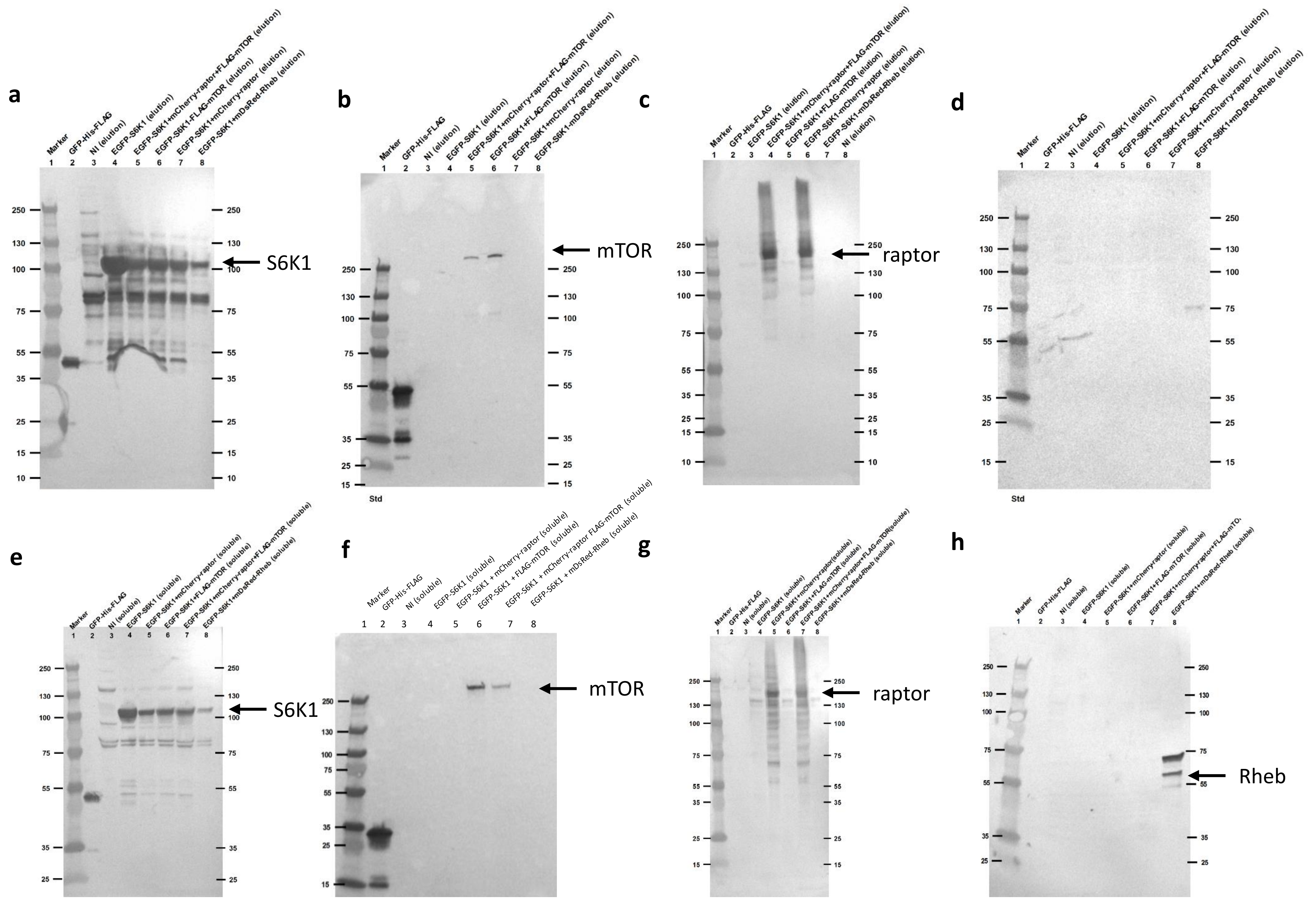

Supplementary Figure S12 – Full lengths gels for Supplementary S5.Western blot of eluted mTORC1 complexes shown for **a** S6K1 using anti-His antibodies. **b** mTOR using anti-FLAG antibodies. **c** raptor using anti-raptor antibodies. **d** Rheb using anti-Rheb antibodies. Western blots of soluble mTORC1 lystates for **e** S6K1 using anti-His. **f** mTOR using anti-FLAG. **g** raptor using anti-raptor. **h** Rheb using anti-Rheb antibodies. Marker, GFP and no transfection (NI) controls are also shown on blots.

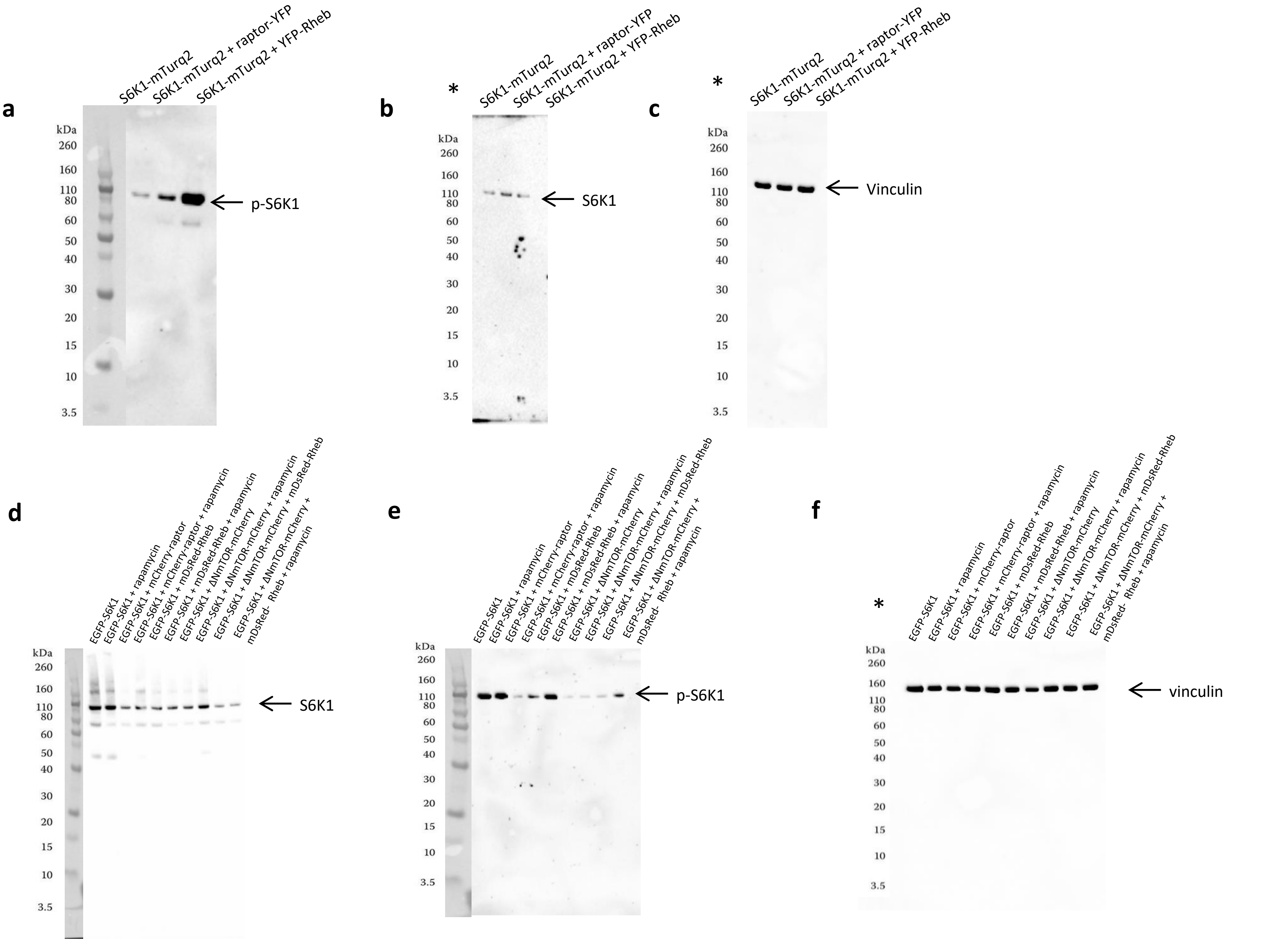

Supplementary Figure S13 – Full lengths gels for Figure 3. **a** WB for phospho-S6K1 using anti-pS6K1 antibody. **b** WB for S6K1 using anti-S6K1. **c** WB for vinculin using anti-vinculin antibody. **d** WB for S6K1 using anti-S6K1 antibody. **e** WB for p-S6K1 using anti-phospho S6K1 antibody. **f** WB for vinculin using anti-vinculin antibody. Unavailable respective colorimetric marker images are indicated (*)

a

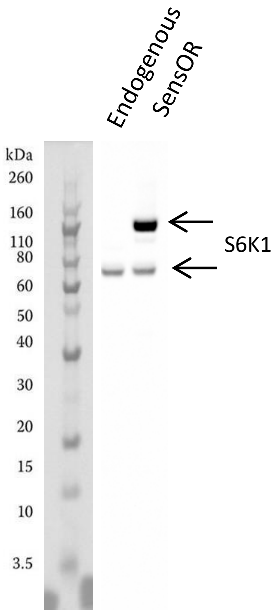

b

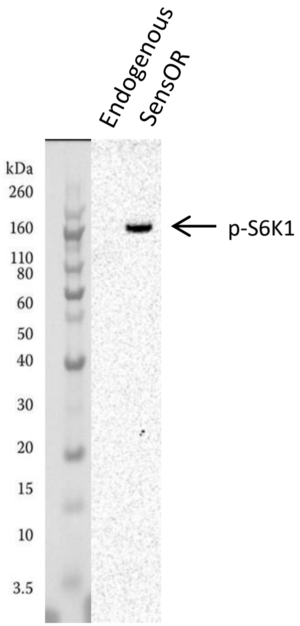

Supplementary Figure S14 – Full lengths gels for Figure 4. **a** WB for S6K1 using anti-S6K1 antibody. **b** WB for p-S6K1 using anti-phosphor-S6K1 antibody.

a

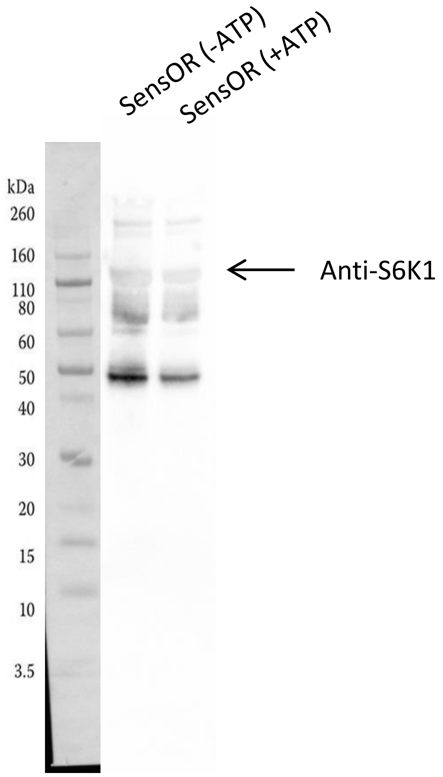

b

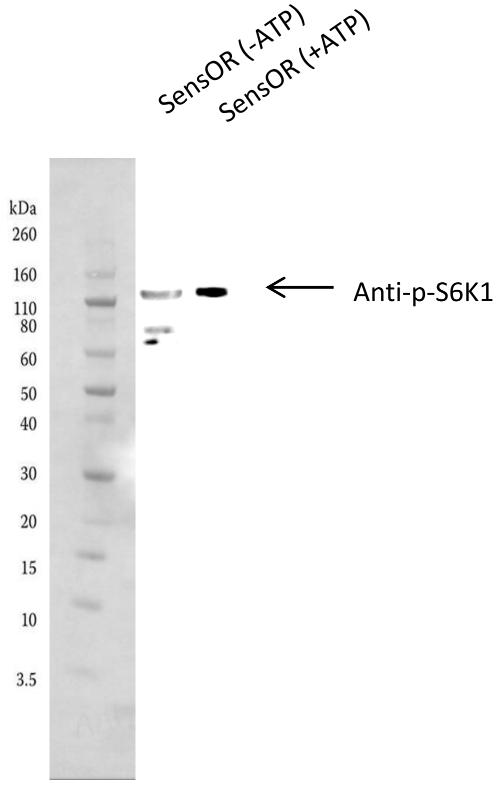

Supplementary Figure S15– Full lengths gels for Supplementary Figure S9ag. **a** WB for S6K1 using anti-S6K1 antibody with and without ATP treatment of SensOR. **b** WB for p-S6K1 using anti-phosphoS6K1 with and without ATP treatment of SensOR. Colorimetric marker images are also shown.

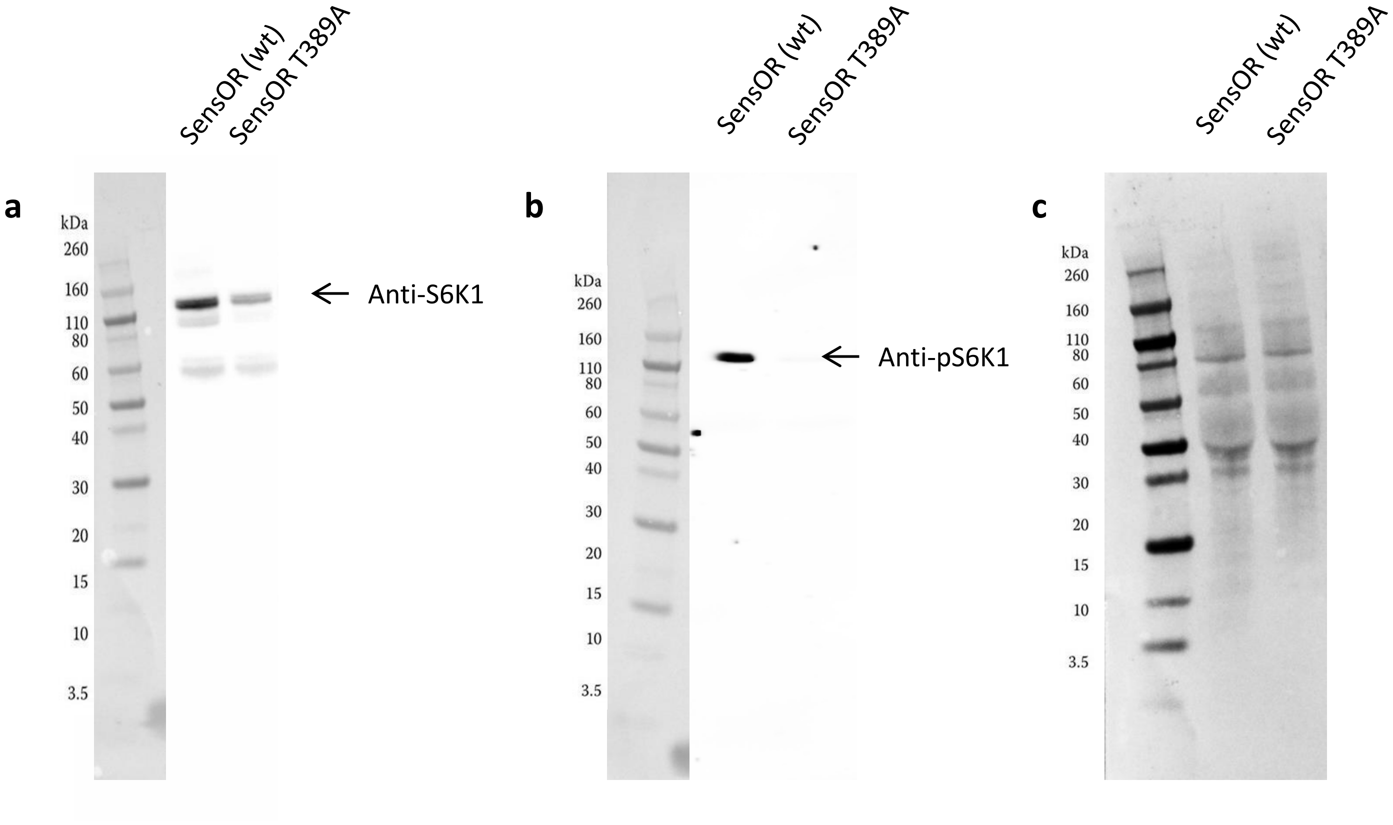

Supplementary Figure S16– Full lengths gels for Supplementary Figure S9b. **a** WB for S6K1 using anti-S6K1 antibody of SensOR wildtype (wt) and SensOR mutant (T389A) and **b** WB for p-S6K1 using anti-phosphoS6K1 antibody of SensOR wildtype (wt) and SensOR mutant (T389A). **c** Ponceau S Stain of blot to check for loading control.
